# Protein moonlighting by a target gene dominates phenotypic divergence of the Sef1 transcriptional regulatory network in yeasts

**DOI:** 10.1093/nar/gkae1147

**Published:** 2024-11-20

**Authors:** Po-Chen Hsu, Tzu-Chiao Lu, Po-Hsiang Hung, Jun-Yi Leu

**Affiliations:** Institute of Molecular Biology, Academia Sinica, 128 Academia Road, Section 2, Taipei 115201, Taiwan, Republic of China; Huffington Center on Aging, Baylor College of Medicine, One Baylor Plaza, Houston, TX 77030, USA; Department of Molecular and Human Genetics, Baylor College of Medicine, One Baylor Plaza, Houston, TX 77030, USA; Department of Genetics, Stanford University Medical School, 300 Pasteur Drive, Stanford, CA 94305, USA; Institute of Molecular Biology, Academia Sinica, 128 Academia Road, Section 2, Taipei 115201, Taiwan, Republic of China

## Abstract

Transcriptional rewiring generates phenotypic novelty, acting as an important mechanism contributing to evolutionary development, speciation, and adaptation in all organisms. The phenotypic outcomes (functions) of transcription factor (TF) activity are determined by the combined effects of all target genes in the TF’s regulatory network. Plastic rewiring of target genes accumulates during species divergence and ultimately alters phenotypes, indicating a TF functional switch. We define this phenomenon as ‘disruptive rewiring’, where the rewiring process disrupts the link between a TF and its original target genes that determine phenotypes. Here, we investigate if ‘complete’ disruptive rewiring is a prerequisite for a TF functional switch by employing chromatin immunoprecipitation sequencing, RNA expression, and phenotypic assays across yeast species. In yeasts where Sef1 targets TCA (tricarboxylic acid) cycle genes, we demonstrate that Sef1 orthologs can promote and inhibit respiratory growth by modulating the moonlighting function of their conserved target, *NDE1*. This modulation occurs without changing the overall association of Sef1 with TCA cycle genes. We propose that phenotypic masking by *NDE1* promotes ‘deceptive’ disruptive rewiring of the Sef1 regulatory network in *Saccharomyces cerevisiae*, thereby potentially constraining future evolutionary trajectories.

## Graphical Abstract

**Graphical Abstract:**
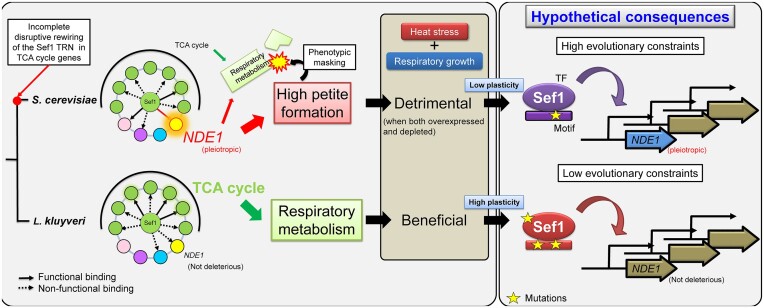

## Introduction

Transcriptional regulation likely evolves in concert with evolutionary development, speciation and the adaptive evolution of organisms (1–4). ‘Transcriptional rewiring’ describes how transcriptional regulation evolves across species by changing the *cis*-regulatory elements in gene promoters or by replacing *trans*-acting regulatory factors in a transcriptional regulatory network (TRN) (5,6), such as in interspecific ‘transcription factor (TF) substitution’ of orthologous target genes or intraspecific ‘regulon handover’ between different TFs (7). As in higher eukaryotes, transcriptional rewiring also occurs in ‘simpler’ microorganisms (7–9). Many studies on yeast species exemplifying diverse scales of rewiring events in multiple cellular processes underlying ribosomal protein production (10–14), sugar metabolism (15–17), nucleotide metabolism (18), mating-type determination (19–21), meiosis/sporulation (22), TCA (tricarboxylic acid) cycle (23) and iron homeostasis (24–26), have been reviewed extensively (6,7,27,28). Theoretically, the ultimate consequence of transcriptional rewiring is a change in the phenotypic outcomes of a TRN (synonymous with a functional switch of the TF) as a result of *cis*-motif and/or *trans*-factor changes that disrupt the regulatory connections between a TF and its original phenotype-determining genes (22). Here, we term this aforementioned phenomenon as ‘disruptive rewiring’ to describe a TF-regulon disconnecting process that eventually leads to TF-phenotype dissociation. In general, qualitatively comparing the phenotypic changes displayed by orthologous TF null mutants between different species represents an intuitive strategy for identifying TF candidates whose networks potentially underwent disruptive rewiring. However, it remains unclear if ‘complete’ disruptive rewiring preceding the TF functional switch is a prerequisite. In particular, we define that a ‘complete’ disruptive rewiring to be: a TF ‘completely’ loses its direct regulation (i.e. a TF no longer binds to the promoters of its target genes or it binds to the promoters but fails to significantly drive the gene expression) on its primary regulon genes which determine a specific phenotype under a specific condition.

**Figure 1.**
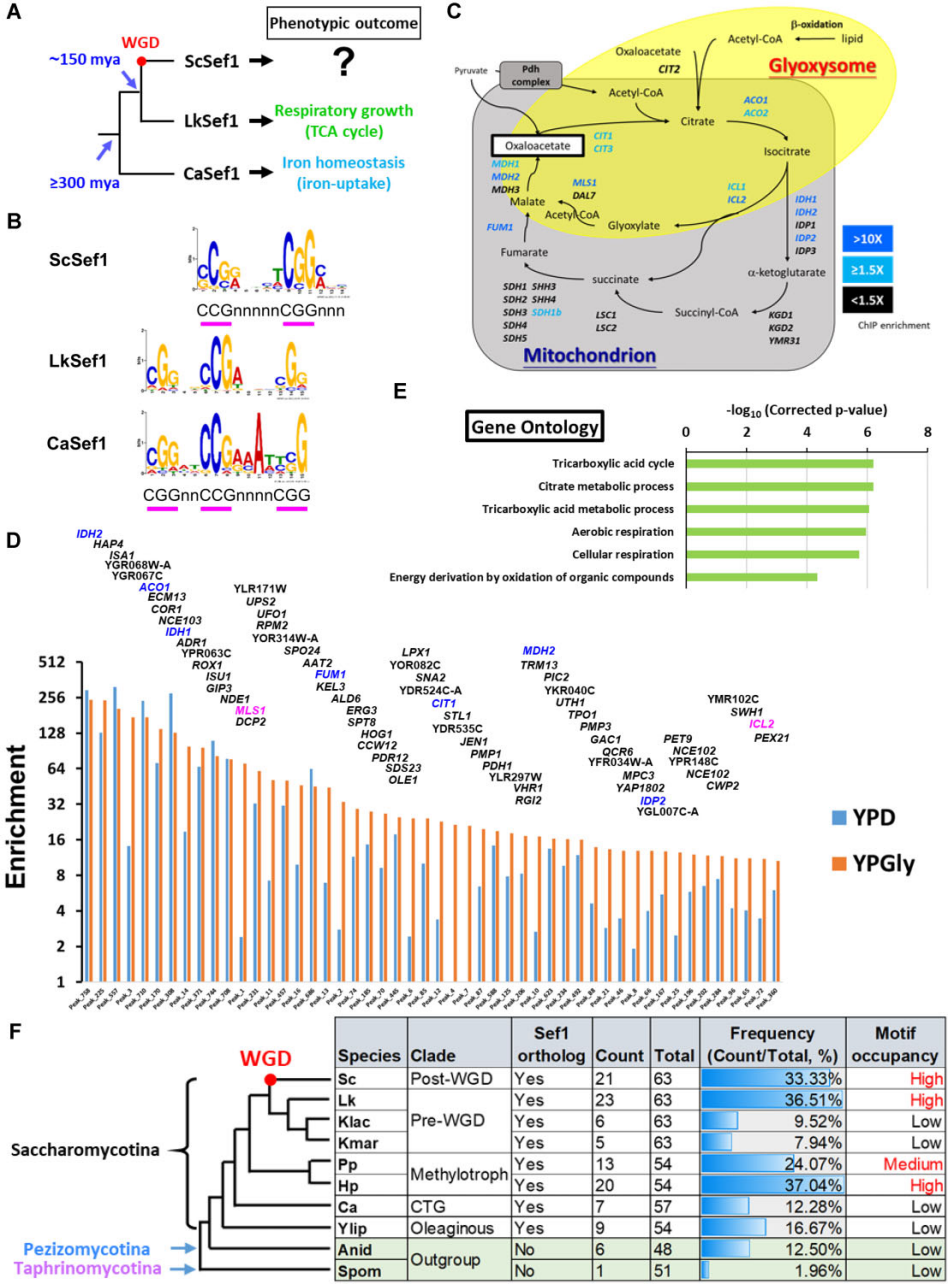
Sef1 in *S. cerevisiae* majorly binds to the promoters of most TCA cycle genes. (**A**) Biological functions of Sef1 orthologs. The Sef1-mediated functions were confirmed by evidence of measurable defective (misregulated) phenotypes of *sef1*Δ mutants compared to each wild type in the protoploid [pre-whole genome duplication (pre-WGD)] yeast *Lachancea kluyveri* (Lk) (23) and the CTG clade yeast *Candidaalbicans* (Ca) (25). Before this study, the biological function of *S. cerevisiae* (post-WGD yeast) Sef1 was generally unclear. WGD, whole genome duplication; mya, million years ago. (**B**) The ScSef1 DNA recognition motif compared to the published consensus binding motifs of LkSef1 (23) and CaSef1 (25). MEME-ChIP was used to generate the position weight matrix (PWM) file of the ScSef1 DNA recognition motif based on the ScSef1 ChIP-seq data. The 250-bp sequences flanking each peak center of the TOP200 enriched Sef1 binding peaks were used in analyses. The LkSef1 and CaSef1 PWM files were taken from a previous work (23). The consensus ‘CGG’ or ‘CCG’ repeats of PWM logos are underlined with pink bars. The accompanying E-value for ScSef1 *de novo* motif prediction is 1.4E–34. (**C**) A schematic diagram of the TCA cycle and glyoxylate shunt. Both genes and pathways are included. Genes targeted by ScSef1 with ChIP-seq enrichment values ≥1.5-fold in either YPD or YPGly conditions are highlighted in blue. Genes with enrichment values <1.5-fold or not targeted by Sef1 are marked in black. (**D**) TOP50 peaks ranked based on the ChIP-seq enrichment values under YPGly conditions. All 645 peaks (Peak_N, x-axis) were ranked based on chromatin immunoprecipitation (ChIP) enrichment values (y-axis) in descending order (orange bars) (also see Supplementary Figure S1B and associated figure legend) and only the TOP50 peaks are shown. The names of target genes assigned for each peak are labeled above each bar, with TCA cycle genes highlighted in blue and glyoxylate shunt genes in pink. (**E**) Gene ontology (GO) analysis of ScSef1 target genes with high-confidence Sef1 binding motifs. There are 123 target genes assigned to 97 ChIP peaks that cover sequences containing at least one FIMO-scanned ScSef1 motif with a *P*-value <0.0001 (also see Supplementary Figure S1D). GO Term Finder and the Bonferroni multiple testing correction in SGD were used, and the enriched GO terms with a corrected *P*-value <0.0001 are shown. (**F**) Occurrence of Sef1 binding motif-like sequences in the promoters of orthologous TCA cycle genes across multiple fungal species, including *S. cerevisiae* (Sc), *L. kluyveri* (Lk), *Kluyveromyces lactis* (Klac), *Kluyveromyces marxianus* (Kmar), *Pichia pastoris* (Pp), *Hansenula polymorpha* (Hp), *C. albicans* (Ca), *Yarrowia lipolytica* (Ylip), *Aspergillus nidulans* (Anid) and *Schizosaccharomyces pombe* (Spom). FIMO was used to scan each 500-bp (−1 to −500 from ATG) promoter of TCA cycle genes for individual matches to each of the three Sef1 motif variants in (**B**) successively. By default, a *P*-value threshold of 0.0001 was used to define the presence of the matched Sef1 motif. The frequency of motifs was calculated as counts/total. ‘Counts’ represents the sum of motif-presence numbers in the promoters of all TCA cycle genes, with multiple matches of one motif on the same promoter being counted only once. ‘Total’ represents the sum of FIMO-scanned numbers using all three Sef1 motif variants for all TCA cycle gene promoters. ‘Low’ motif occupancy was defined according to the scan results from two species (Anid and Spom) lacking a Sef1 ortholog and *C. albicans* whose Sef1 does not regulate TCA cycle genes (25). The motif occupancy similar to that scanned from the promoters of *L. kluyveri* TCA cycle genes was defined as ‘high’, whereas similar to or lower than that from *C. albicans* was defined as ‘low’ (23). A particular ‘gain and loss’ pattern of Sef1 motifs was apparent for the promoters of TCA cycle genes among *Saccharomycotina* yeasts.

To address this issue, we have characterized functional switch events across yeasts in a TF, Suppressor of essential function 1 (Sef1), the orthologs of which evidence at least two (Figure 1A) and possibly more evolutionary trajectories (23). However, the primary function of Sef1 in *Saccharomyces cerevisiae* (ScSef1) remains unclear. Therefore, we used chromatin immunoprecipitation sequencing (ChIP-seq), gene expression and phenotypic assays to investigate ScSef1 and uncovered pseudo-rewiring in the *S. cerevisiae* Sef1 TRN, the phenotypic outcomes of which are masked (dominated) by a conserved target gene, *NDE1*. The species-specific moonlighting function of Nde1 increases the frequency of petite formation in the yeast population. Yeast petite phenotype results from mutations in the mitochondrial genome (ρ^–^), loss of mitochondria (ρ^0^) or mutations in the nuclear genome, all of which cause mitochondrial dysfunction (29). Consequently, a Nde1-induced higher frequency of petite individuals in the *S. cerevisiae* population reduces population fitness, especially under heat-stressed conditions. Moreover, we defined the phenomenon in which a target gene exerting a strong phenotypic impact (in our case, a deleterious effect) masks the phenotypic outputs of other target genes in a TRN as ‘phenotypic masking’. Such ‘phenotypic masking’ by different phenotype-determining genes of the original regulon may constrain evolution of the upstream transcriptional regulator and the corresponding TRN. Our study not only demonstrates that a TF can switch function before disruptive rewiring has been completed, but also reveals that evolution of moonlighting functions of a conserved target gene can underlie interspecific functional changes of a TF.

## Materials and methods

### Genome resources

Details of the genome sequences and annotations used in this study are listed in Supplementary Table S8. Here, only BioProject accession numbers are shown: *S. cerevisiae* (PRJNA260311, PRJNA324291 and PRJNA43747); *L. kluyveri* (PRJNA1445); *K. lactis* (PRJNA13835); *K. marxianus* (PRJDA65233); *P. pastoris* (PRJEA37871); *H. polymorpha* (PRJNA60503); *C. albicans* (PRJNA10701); *Y. lipolytica* (PRJNA295780); *A. nidulans* (PRJEA40559); and *S. pombe* (PRJNA13836). The *SEF1* and *NDE1* orthologs were identified based on each genome annotation and then confirmed by a reciprocal BLASTp strategy. Orthologs of TCA cycle gene families were confirmed by using OrthoVenn2 (30) with default settings. To reduce the complexity of paralogy assignments, *L. kluyveri* protein sequences were used as a reference due to its pre-WGD genome lacking ohnologs.

### Strain and plasmid construction

To avoid confusion, we have adopted the term ‘wild type’ to describe the parental genotypes used in our study, each of which may carry auxotrophic mutations or specific genetic manipulations, and they acted as reference strains for all comparative experiments unless otherwise stated. The yeast strains, plasmids and primers used in this study are listed in Supplementary Table S9. All DNA fragments used in cloning were amplified by polymerase chain reaction (PCR) using Phusion High-Fidelity DNA polymerase (F530L, Thermo Fisher Scientific, USA). The PCR products and restriction digested products were purified by PCR cleanup or gel extraction using a QIAquick^®^ PCR Purification Kit (28 106, QIAGEN, Germany) or a QIAquick^®^ Gel Extraction Kit (28 706, QIAGEN, Germany). DNA cloning into plasmids was achieved by using ligation (T4 DNA ligase, M180A, Promega, USA) or using an In-Fusion^®^ HD Cloning Kit (639 650, ClonTech by Takara, Japan). All plasmids were purified using a Presto^TM^ Mini Plasmid Kit (PDH300, Geneaid, Taiwan). Successful strain constructions were confirmed by genomic DNA extraction, as described previously (26), followed by PCR diagnostics using a homemade Taq DNA polymerase for genotyping.

For gene deletions, the DNA fragments of each deletion module consisting of a selection marker flanked by 5′ and 3′ sequences homologous to the target locus were created by overlap-extension-PCR (31). Deletion of *NDE1* in *S. cerevisiae* is the only exception, in which the deletion KanMX4 cassette was directly PCR-amplified from the genomic DNA of the *nde1*Δ strain of the yeast deletion collection. The *S. cerevisiae* genes deleted in this study are: *SEF1* (YBL066C); *NDE1* (YMR145C); *AFT2* (YPL202C); *HRD3* (YLR207W); and *BRE5* (YNR051C). The *L. kluyveri* gene deleted in this study is *NDE1* (SAKL0E05302g).

For lexA, TAP and VP16 tagging of *S. cerevisiae* Sef1, we used a modified (23) SAT1 flipper method (32). To produce lexA tagging plasmid, the entire 3443-bp open reading frame (ORF) without STOP codon of *SEF1* was ligated into the pSFS2A-lexA-5 vector between the *Kpn*I and *Apa*I restriction sites, and the 325-bp 3′ untranslated region (UTR: terminator, +72 to +396 from the STOP codon) sequence of *SEF1* was ligated between the *Sac*II and *Sac*I restriction sites, thereby generating pSFS2A-ScSef1-lexA-t-7. To produce TAP tagging plasmid, the TAP sequence was amplified from pSFS2A-TAPSacIMut-1 and ligated into the pSFS2A-ScSef1-lexA-t-7 vector between the *Apa*I and *Xho*I restriction sites to replace the lexA sequence, thereby generating pSFS2A-ScSef1-TAP-4. To produce VP16 tagging plasmid with the *CaMAL2*p-driven SAT1-FLIP cassette, the VP16 sequence was amplified from pC2HP and cloned into the pSFS2A-ScSef1-lexA-t-7 vector between the *Apa*I and *Xho*I restriction sites to replace the lexA sequence using In-Fusion^®^, thereby generating pSFS2A-ScSef1-VP16-t-N1. To produce VP16 tagging plasmid with the *ScGAL1*p-driven SAT1-FLIP cassette, we first created pSFS4A-1 vector by replacing the *CaMAL2* promoter on pSFS2A with the *ScGAL1p* promoter using In-Fusion^®^. Then the *ScSEF1-VP16* fragment amplified from pSFS2A-ScSef1-VP16-t-N1 was ligated into the pSFS4A-1 vector between the *Kpn*I and *Xho*I restriction sites, and the 325-bp 3′-UTR (terminator, +72 to +396 from the STOP codon) sequence of *SEF1* was ligated between the *Sac*II and *Sac*I sites, thereby generating pSFS4A-ScSef1-VP16-t-N2. The *Kpn*I*/Sac*I-digested fragments from the three tagging plasmids were used to transform yeast cells. *SEF1* was tagged with TAP or VP16 in *S. cerevisiae* wild type, whereas the lexA-tagging fragment of *SEF1* was transformed into the one-hybrid reporter strain W303lexAOPLacZ-1.

To create the lexA-only control strain for ScSef1 one-hybrid assays, a 271-bp DNA fragment of the *ScSEF1* promoter (−271 to −1 from ATG) was ligated into the pSFS2A-lexA-5 vector between the KpnI and ApaI restriction sites to create a PCR template (Notably, it is a transient PCR template during strain construction so that we did not keep this plasmid in our plasmid list) of *ScSEF1p-lexA* fragments. A 526-bp DNA fragment of the 5′ flanking region (Chr02:341 111_341 636), the *ScSEF1p-lexA* fragment, the KanMX6 drug maker (amplified from plasmid pFA6a-KanMX6) and the 628-bp 3′ flanking region (Chr02:342 102_342 729) were PCR-fused and integrated into the genomic neutral site (33), which locates between the genes YBR053C and *YRO2* (YBR054W) on chromosome 2 of the one-hybrid reporter strain W303lexAOPLacZ-1.

To create *S. cerevisiae* one-hybrid reporter strains, an 883-bp DNA fragment of the *HIS3* 5′ flanking region (YOR202W, −10 to −892 from ATG), the lexA operator-controlled lacZ reporter with an upstream *URA3* marker (*ScURA3-GAL1p^8XLexAOP^-lacZ*; amplified from the pSH18-34 vector), and the 593-bp 3′ flanking region of *HIS3* (+13 to +605 from the STOP codon) were PCR-fused and integrated into the *his3-11* locus of the W303 strain.

To create the promoter-LacZ plasmids, the sequences of wild-type *NDE1* (YMR145C) and Sef1-motif-mutated promoters were PCR-amplified and cloned into the reporter plasmid pRS41H-LacZ-1 between the *Kpn*I and *Apa*I restriction sites using In-Fusion^®^. The Sef1-binding motif on the *NDE1* promoter was mutated by overlap-extension-PCR-based site-specific mutagenesis (34). The promoter-LacZ strains were created by transforming JYL03, W303ScSef1NA1 and W303ScSef1VP16NB1-4 with the corresponding plasmids.

To create the *NDE1* ortholog-expressing plasmids, DNA fragments of the *ScNDE1* promoter (−1 to −1007 from ATG) and terminator (+1 to +402 from STOP codon) were cloned into the pRS41H vector using In-Fusion^®^ between the *Kpn*I and *Apa*I restriction sites and the *Not*I and *Sac*I sites, respectively, thereby generating pRS41H-ScNDE1p-t-2. The *ScNDE1* and *LkNDE1* (SAKL0E05302g) ORFs were cloned into the pRS41H-ScNDE1p-t-2 vector between the *Apa*I and *Not*I sites using In-Fusion^®^ to generate pRS41H-ScNDE1p-ScNDE1-t-1 and pRS41H-ScNDE1p-LkNDE1-t-1, respectively. To create the *ScNDE1*-expressing plasmids driven by the Sef1 motif-mutated *ScNDE1* promoter, the *ScNDE1pMut* fragments were PCR-amplified from the pRS41H-ScNDE1pMut-LacZ-4 vector and cloned into the *Kpn*I and *Apa*I digested pRS41H-ScNDE1-t vector (an intermediate product not inventoried in the plasmid list) using In-Fusion^®^, thereby generating pRS41H-ScNDE1pMut-ScNDE1-t-6.

All yeast transformations were performed by using electroporation, as described previously (23). The URA^+^ transformants of the one-hybrid reporter strain were selected by growth in SC − Ura. All strains transformed with pRS41H or its derivative plasmids were selected and maintained in YPD (yeast extract-peptone-dextrose) + HGB (Hygromycin B) or YPGly (yeast extract-peptone-glycerol) + HGB. Notably, since the *C. albicans MAL2* promoter on the SAT1-FLIP cassette is leaky in *S. cerevisiae*, the *S. cerevisiae* SAT1-FLIP transformants could be selected and propagated in either YPD + Nou10 (10 μg/ml Nourseothricin) or YPD + Nou (75 μg/ml Nourseothricin) agar plates during strain construction procedures. The integrated SAT1-FLIP cassette does not support efficient growth of *S. cerevisiae* cells in liquid broth with Nourseothricin. To pop out the integrated *CaMAL2*p-driven SAT1-FLIP cassette in the lexA or TAP-tagged strains, the *MAL2* promoter was induced by YPMal. In brief, cells were inoculated into 3 ml YPMal and grown at 28°C. Overnight-cultured cells were then plated onto YPMal agar plates and incubated at 28°C for 3 days. Single colonies were then screened for Nourseothricin resistance by streaking them on both YPD and YPDNou10 plates, and the Nou-sensitive strains were picked for PCR diagnostics. To pop out the *S. cerevisiae GAL1* promoter-driven SAT1-FLIP cassette in the VP16-tagging strains, the cells of pre-popout strains pre-grown in YPRaf at 28°C for 2 days were diluted 100-fold into 5 ml YPGal medium and grown at 28°C for 2 days. Then the post-cultured cells were streaked on YPGal plates and grown at 28°C for 3 days to obtain single colonies for screening of Nourseothricin-sensitive clones.

To derive petite clones from yeast strains, the tested strains were inoculated in YPD-based media and grown overnight at 28°C. The cells were then spread on adenine-limiting YPD plates at a density of 100–300 colony-forming units (CFU)/plate and incubated for 3 days. Petite colonies are small and white, unlike ‘grande’ colonies (also see the petite formation assays described below in the ‘Phenotypic assays’ section). Only completely white, round and smooth colonies without red sectors were picked and double-checked for lack of respiratory growth by growing them at 28°C for 3 days on YPGly plates (on which pure petite clones cannot grow).

To create strains used for ScSef1 mediated ChIP-qPCR (chromatin immunoprecipitation-quantitative polymerase chain reaction) and ScSef1 motif carrying plasmid pull-down assays, plasmids pRS41H-ScNDE1p1007-LacZ-2 with a 1007-bp wild-type *NDE1p* promoter and pRS41H-ScNDE1pMut-LacZ-4 carrying a Sef1 binding site-mutated *NDE1p* promoter were separately transformed into the ScSef1-TAP strain (W303ScSef1TAPNB2-1).

### Media, important chemicals and growth conditions

All media and chemicals used in this study are listed with abbreviations in Supplementary Table S9. All agar plates contained 2% agar. Media were sterilized by autoclaving. All cultures in culture tubes were grown on a drum roller rotating at 65–80 rpm, while cultures in flasks were placed on an orbital shaker rotating at 180 rpm. The normal growth temperature was 28–30°C. Heat stress was applied at 37–39°C, as indicated in the experiments. Notably, due to our usage of *S. cerevisiae* strains with a W303 background (adenine auxotroph), all YP-based media were additionally supplemented with 0.02% adenine and tryptophan to avoid forming red cells, except for the adenine-limiting YPD medium (no additional adenine and tryptophan) used to distinguish petite colonies from non-petite colonies.

### Phenotypic assays

For the spot assays, cells grown in YPD or YPD + HGB (for plasmid selection) overnight at 28°C were harvested by centrifugation (Eppendorf 5810R centrifuge; A-4–62 rotor; 1500 *g*; 3 min; 25°C) and serially diluted to 10^7^–10^3^ CFU/ml with sterile ddH_2_O. Each dilution was spotted onto agar plates (5 μl/spot, ∼10^5^–10^1^ CFU/spot) and incubated at 28°C or heat-stressed temperatures as indicated. The plates were scanned using an Epson Perfection V800 Photo scanner and images were recorded after 3 days or as otherwise indicated.

For the petite-prone assays under heat stress, cells grown from one single colony in 10 ml YPGly at 28°C for 20.5 h were harvested and subcultured into 5 ml YPD at a cell density of 0.2 OD_600_/ml. Petite formation was induced by incubating the cultures at 39°C with shaking at 65 rpm. At 0, 5.5, 24 and 48 h, 1 ml of culture was harvested, diluted and spread on YPD plates. For the 0-, 5.5- and 24-h samples, cells were spread at a density of 10^2^ CFU/plate, whereas 10^3^ CFU/plate was used for the 48-h samples. TTC (2,3,5-triphenyl tetrazolium chloride) reduction assays were performed to visualize the petite and grande colonies grown at 28°C for 3 days. In brief, colonies on the plates were overlaid with 20 ml melted TTC agar [0.1% TTC, 2% agar in phosphate-buffered saline (8 g/l NaCl, 0.2 g/l KCl, 1.44 g/l Na_2_HPO_4_, 0.24 g/l KH_2_PO_4_, pH 7.4)] and the agar was allowed to solidify for 30 min at room temperature. The overlaid plates were incubated at 28°C for red color development for ≥2.5 h. The inability of petite cells to reduce TTC generates white colonies, in contrast to the red grande colonies of non-petite cells resulting from the reduced products of TTC. The petite frequency was calculated by counting the numbers of while colonies over total colonies from five technical repeats.

For the basal petite formation assays, to reduce phenotypic variation of plasmid-bearing strains with higher petite-prone activities, cells from −80°C stock were first grown on YPD + HGB plates at 28°C for 3 days and then patched to YPGly + HGB plates for growth for 5 additional days to maximally select against petite cells. The selected cells were subsequently inoculated into 5 ml YPD + HGB or YPGly + HGB and incubated at 30°C to allow petite cell accumulation. After 20 h, the cells were track-spread on adenine-limiting YPD plates and incubated for 3 days. The inability of *ade2^–^* petite cells to accumulate red pigments in vacuoles due to the blocked adenine biosynthesis pathway generates white colonies in response to adenine depletion, contrary to the red grande colonies of non-petite cells (35,36). The petite frequency was calculated by counting the numbers of while colonies over total colonies from three technical repeats.

For the competitive desiccation assays, a single colony of each tested strain was inoculated in 5 ml YPD and grown at 28°C for 20 h. Cells were then harvested and resuspended in 1 ml sterile ddH_2_O. Equal amounts (0.5 OD_600_) of the reference wild-type strain (drug-sensitive) and the test strain (G418- or Nou-resistant) were mixed in 1 ml sterile ddH_2_O in a 1.5 ml tube. The desiccated sample was prepared by spinning down cells, removing supernatants, sealing the opened tube with a sterile breathable sealing film (BF-400-S, AXYGEN, USA), and air-drying the cell pellets at 28 or 39°C for 24 h. Control samples were prepared by retaining the supernatants and keeping the tubes capped. After 24 h, the cells were rehydrated in 1 ml sterile ddH_2_O for 1 h, track-spread on YPD plates and incubated at 28°C for 2 days. The relative viability of drug-resistant and -sensitive cells was calculated by CFU counting on these YPD plates and then on replicated YPD + drug plates from three technical repeats after 1 day of incubation at 28°C.

For the growth curve assays, cells grown overnight in YPD at 28°C were inoculated into 120 μl YPD or YPGly medium in a 96-well plate (Tissue Culture Testplate 96 wells F-bottom, 92 096, TPP) at a cell density of 0.005 OD_600_/ml at indicated temperatures. Cell growth was measured at OD_595_ in ‘2 × 2 multiple reads per well’ mode every 12 min using a Tecan plate reader (Infinite 200 PRO, Tecan, Switzerland). Tecan software Magellan Version 7.2 was used for data acquisition and analyses. For the Magellan method, we selected the plate definition ‘[TPP96ft]-Tecan Plastic Products AG6 Flat Transparent’. Each 12-min cycle comprised 3 min reading, 1 min shaking, 3 min standing, 1 min shaking, 3 min standing and 1 min shaking. For the glutathione-replenishing media, 2.5 or 20 mM reduced L-glutathione or N-Acetyl-L-cysteine was added to the YPD or YPGly medium.

For the viability assays in the continuous cultures, cells grown in 5 ml YPD + HGB medium at 28°C for 20 h were subcultured into 10 ml YPGly + HGB medium and incubated continuously at 28°C for 8 days. On days 1, 6 and 8, cells were collected, serially diluted and track spread onto YPD plates. The plates were incubated at 28°C for 3 days. Changes in viability with time were calculated by CFU counting from six technical repeats relative to initial viability at day 1, which was defined as 100%.

### β-Galactosidase assays

The LacZ expression levels for the one-hybrid assays and promoter assays were quantified using liquid β-galactosidase assays, as described previously (26), with some modifications (23). In brief, early log-phase (0.5–1.0 OD_600_/ml) cells cultured in YPD or YPGly media were assayed. All subcultures were initiated with 0.2 OD_600_/ml of inoculum from overnight-grown cells and subcultured for 4.5 h. Notably, the promoter-activity strains were cultured in the presence of 200 μg/ml HGB to select the plasmids. LacZ levels were calculated from three technical repeats and are displayed as Miller units.

### ChIP and ChIP-seq analyses

W303ScSef1TAPNB1-1 (*ScSEF1-TAP*) cells were grown overnight in YPD medium at 30°C and subsequently diluted into YPD or YPGly medium at a cell density of 0.2 OD_600_/ml. After 4.5 h of incubation at 30°C, cultures of early log-phase cells (1 OD_600_/ml in YPD or 0.4 OD_600_/ml in YPGly) were fixed with 1% formaldehyde at 25°C for 15 min with shaking at 180 rpm and then quenched with 125 mM glycine at 25°C for 10 min with shaking at 180 rpm. All subsequent steps were performed in an ice-cold or 4°C environment. Cells were harvested, washed twice with TBS [20 mM Tris-Cl (pH = 7.5), 150 mM NaCl] and stored at −80°C until use. For YPD-grown samples, 1500 OD_600_ cells per sample were harvested, whereas 500 OD_600_ cells per sample were harvested for YPGly-grown samples.

For ChIP experiments, lysis of fixed cells by bead beating, DNA shearing using a Bioruptor (Diagenode, USA), immunoprecipitation with Dynabeads^®^ Pan Mouse IgG (11 041, human anti-mouse IgG, Invitrogen by Thermo Fisher Scientific), RNase A treatment, Proteinase K treatment, de-crosslinking at 65°C for 16 h and ChIP DNA purification using a QIAquick^®^ PCR Purification Kit (28 106, QIAGEN, Germany) were all performed as described previously (23). Notably, the 1500 OD_600_ YPD-grown samples were split into three 500 OD_600_ technical repeats for ChIP experiments due to a capacity limitation of the ChIP protocol. The final ChIP DNA from three cognate technical repeats was pooled and concentrated to represent one biological repeat. DNA concentrations were measured using a Qubit™ dsDNA HS Assay Kit (Q32854, Invitrogen by Thermo Fisher Scientific).

ChIP-seq analysis was performed on four biological replicates of YPD-grown cells and three biological replicates of YPGly-grown cells. The average fragment length of sonicated DNA was 150–1000 bp and it was assessed using a Bioanalyzer 2100 instrument (Agilent Technologies) with a High Sensitivity DNA Kit (Agilent Technologies). For each condition, libraries were prepared from >3 ng of ChIP-DNA or input DNA. The library preparation, single-read sequencing (75 bp) of the libraries at a depth of ∼40 million reads (∼200-fold genome coverage) and read trimming were performed as described previously (23). Mapping of the trimmed reads to the *S. cerevisiae* S288C genome (R64-2-1), peak calling, and peak merging were conducted as described previously (23). Only peaks detected in at least three biological repeats under either condition (645 peaks out of 785 total peaks) were used for downstream analyses. Notably, the library from biological repeat no. 3 of YPGly-grown samples was paired-end sequenced (75 bp × 2) separately due to some mishandling. Consequently, the paired-end reads of this sample were merged and analyzed together with the other single-read samples, with final ChIP peaks not varying from the other two biological repeats.

For peak-to-gene assignments, peaks were assigned to specific genes based on the location of each peak center. Only peaks with centers located within the region −2000 to +200 from the ATG of the nearest target genes were assigned. A total of 305 (47%) peak centers were located at a distance of ≤ 500 bp from ATG, 189 peak centers were located at a distance of between 500 and 1000 bp from ATG, 99 peak centers were located at a distance of between 1000 and 2000 bp from ATG, and 52 peak centers were located at a distance of >2000 bp from ATG (Supplementary Table S1). Overall, of 645 peaks, 593 were assigned to target genes. To increase the accuracy of target gene identification, at least three biological replicates were performed for each condition. Only genes with Sef1-binding peaks in all three replicates were considered *bona fide* targets, especially for weakly bound targets.

### ChIP-qPCR analyses

DNA samples were prepared in the same way as described above in the ChIP-seq experiments. The strains, W303ScSef1TAP-pRS41H-ScNDE1p1007-LacZ-1 and W303ScSef1TAP-pRS41H-ScNDE1pMut-LacZ-1, were used. The final ChIP and input DNA concentrations were measured using a Qubit™ dsDNA HS Assay Kit (Q32854, Invitrogen by Thermo Fisher Scientific). qPCR (quantitative polymerase chain reaction) was performed using the model QuantStudio^TM^ 12 K Flex Real-Time PCR System (Applied Biosystems by Thermo Fisher Scientific). A total of 0.01 and 0.5 ng per repeat of ChIP and input DNA were used for qPCR, respectively. Primers used for qPCR are listed in Supplementary Table S9. The primers recognizing DNA sequences flanking the Sef1 motif were used to detect Sef1 binding. The qPCR primers recognizing the telomere end at the right arm of chromosome 6 were used as the unbound background for normalization (ΔCt). The fold enrichment of ChIP relative to each input was calculated using the 2^−ΔΔCt^ method from three technical repeats.

### RNA extraction and gene expression microarray analyses

Approximately 10–15 OD_600_ of early log-phase cells (1.6 OD_600_/ml in YPD and 0.6 OD_600_/ml in YPGly) grown for 5.5 h under the indicated media at 30°C from subcultures starting with 0.2 OD_600_/ml of inoculum were harvested. Total RNA was extracted using the phenol-chloroform method and treated with a TURBO DNA-free^TM^ kit (AM1907, Ambion, Invitrogen by Thermo Fisher Scientific), as described previously (23) but with the minor modification of lysing the cells with a FastPrep-24™ 5G Homogenizer (MP Biomedicals™, USA) (37). RNA quality was checked using a Bioanalyzer 2100 instrument (Agilent Technologies, USA) with an RNA 6000 Nano LabChip kit (Agilent Technologies), and the RNA was stored at −80°C until use.

RNA abundance was quantitated from four biological replicates using single-color Agilent Yeast Gene Expression Microarray technology (Yeast V2 Gene Expression Microarray 8 × 15K, G4813A-016322, Agilent Technologies). Alexa dye-labeled cDNA synthesis, purification, array hybridization and washing, image scanning and image feature extraction were performed as described previously (23).

For microarray data analyses, we used GeneSpring GX 11.5 software (Agilent Technologies). Raw RNA expression levels were normalized to the 75th percentile intensity. Genes with raw intensity <200 under all conditions were omitted from our analyses. Probability scores were calculated with a default unpaired *t*-test of the ratio of median values from four biological replicates. Differences in gene expression with adjusted *P*-values <0.05 and a fold-change of at least 1.5 were considered significantly different. Benjamini–Hochberg corrections for *P*-values were applied. All results in the GeneSpring output format are presented in Supplementary Tables S2–S7.

### MEME-ChIP analyses for motif discovery

Peak regions were defined as ±125 bp from each peak center (total 250 bp). The 250-bp peak sequences of the top 200 peaks bound by *S. cerevisiae* Sef1 and enriched under the YPGly condition were submitted to MEME-ChIP version 5.1.0 (http://meme-suite.org/tools/meme-chip) (38) for motif discovery using the following settings: Minimum width  =  6, Maximum width  = 30, Count of motifs  = 10, Motif site distribution  = zero or one occurrence per sequence. All other parameters were the default settings.

### FIMO (Find Individual Motif Occurrences) analyses for motif scanning

Motif 1 identified from MEME-Chip, as described above, was adopted as the *S. cerevisiae* Sef1 binding motif and was submitted to FIMO version 5.5.5 (39) built into the MEME-suite (http://meme-suite.org/tools/fimo) to scan the 645 peak sequences bound by ScSef1. As a preliminary analysis, a *P*-value of <0.1 was applied to obtain a full list of scanned motifs. The *P*-value represents the probability of a random sequence of the same length as the motif matching that position of the sequence with a score at least as good as the motif. Subsequent manual filtering by changing the *P*-value cut-off from 0.1 to 0.0001 identified high-confidence motifs. The number of motifs identified based on different *P*-value cut-offs is also shown in Supplementary Figure S1D.

### GO analyses

GO Term Finder (version 0.86) and the Bonferroni multiple testing correction in SGD (*Saccharomyces* Genome Database, http://www.yeastgenome.org) were used to analyze the 123 ScSef1 target genes with high-confidence Sef1 binding motifs. The GO terms with corrected *P*-values <0.0001 were considered significantly enriched.

### Statistical analyses

Details of statistical analyses are presented in the main text or corresponding figure legends. Statistical significance tests were carried out using the unpaired Student’s *t*-test in Excel 2016, online one-way ANOVA (analysis of variance) followed by Tukey’s multiple comparisons *post hoc* test (https://houssein-assaad.shinyapps.io/TableReport/) (40), or the default functions packaged in each analysis tool.

## Results

### Sef1 orthologs in *S. cerevisiae* and *L. kluyveri* target multiple TCA cycle genes

Previously, we demonstrated that a *S. cerevisiae sef1*Δ mutant displays no deficiency in iron-depleted or respiratory growth (23), and similar observations were also reported by Chen *et al.* (25) and Gerwien *et al.* (24). These results indicate that ScSef1 functions differently from its orthologs in *C. albicans* and *L. kluyveri* and implying that ScSef1 has potentially been rewired to regulate distinct phenotype-determining gene(s) (Figure 1A). Therefore, we used ChIP-seq to identify Sef1 binding sites across the *S. cerevisiae* genome, allowing us to determine the ScSef1 binding motif that presents a typical ‘CGG’ feature of the zinc cluster TF family, consistent with the known motifs of its orthologs (Figure 1B). Notably, although ScSef1 prefers to recognize dimeric CGG motif with a 5-mer spacing, our previous study demonstrated that ScSef1 can still bind to the trimeric CGG motif with a 2- and a 4-mer spacing to partially rescue the growth defects of *sef1*Δ in *L. kluyveri* cells (23), suggesting that the functions of ScSef1-type dimeric CGG motif and Lk/CaSef1-type trimeric CGG motifs do not change completely (Figure 1B). Moreover, we detected a total of 645 ScSef1 binding targets, corresponding to 678 genes, under either fermentative (YPD) or respiratory (YPGly) growth conditions (Supplementary Table S1 and Supplementary Figures S1A and B). Surprisingly, 14 ScSef1 ChIP peaks were assigned to 12 of a total of 28 TCA cycle genes (Supplementary Figure S1C and Figure 1C). Moreover, not only does ScSef1 exhibit a higher binding preference for TCA genes relative to other targets of the entire genome {Figure 1D and Supplementary Figure S2A [χ^2^ (1, N = 645) = 43.7473, *P* <0.00001]}, but GO terms linked to the TCA cycle are enriched among the ChIP target genes identified as possessing the high-confidence consensus Sef1 binding motif (Supplementary Figure S1D and Figure 1E).

To explore the possible diverse evolutionary trajectories of Sef1, we focused on potential Sef1-mediated regulation of TCA cycle genes across multiple fungal species. The orthologous Sef1 binding motifs from *S. cerevisiae* (this study), *L. kluyveri* (23) and *C. albicans* (25) were used to scan the promoters of TCA cycle genes across all those species (Figure 1B and F). To maximally cover possible motif variants across species, we combined the scanning results from these three motifs to calculate the species-specific Sef1-binding motif occupancies (frequencies) for these genes. Compared to the two outgroup species lacking a Sef1 ortholog (*A. nidulans* and *S. pombe*) and *C. albicans* whose Sef1 is known to not regulate TCA cycle genes (25) (i.e. thus presenting low Sef1 binding motif occupancy and defined as the background of motif occurrence), the most parsimonious model indicates that Sef1 motif gain arose in the common ancestor of methylotrophic, pre-whole genome duplication WGD and post-WGD yeasts, followed by loss of Sef1 motifs during divergence of *L. kluyveri* and *Kluyveromyces* species (Figure 1F). These results support that Sef1 orthologs and their TRNs have multiple evolutionary trajectories. Moreover, *S. cerevisiae* and *L. kluyveri* share ∼15% of Sef1 target genes (Supplementary Figure S2B). This proportion is typical for shared target genes regulated by functionally conserved TF between pre-WGD and post-WGD yeasts, e.g. ∼13% of target genes shared by sporulation-regulating Ntd80 orthologs in *S. cerevisiae* and *K. lactis* (22). Thus, despite some differences in the Sef1-targeted TCA cycle genes of *L. kluyveri* and *S. cerevisiae* (23), TCA cycle genes remain the major binding targets of ScSef1.

### Pervasive non-functional binding of Sef1 to the promoters of TCA cycle genes in *S. cerevisiae*.

**Figure 2.**
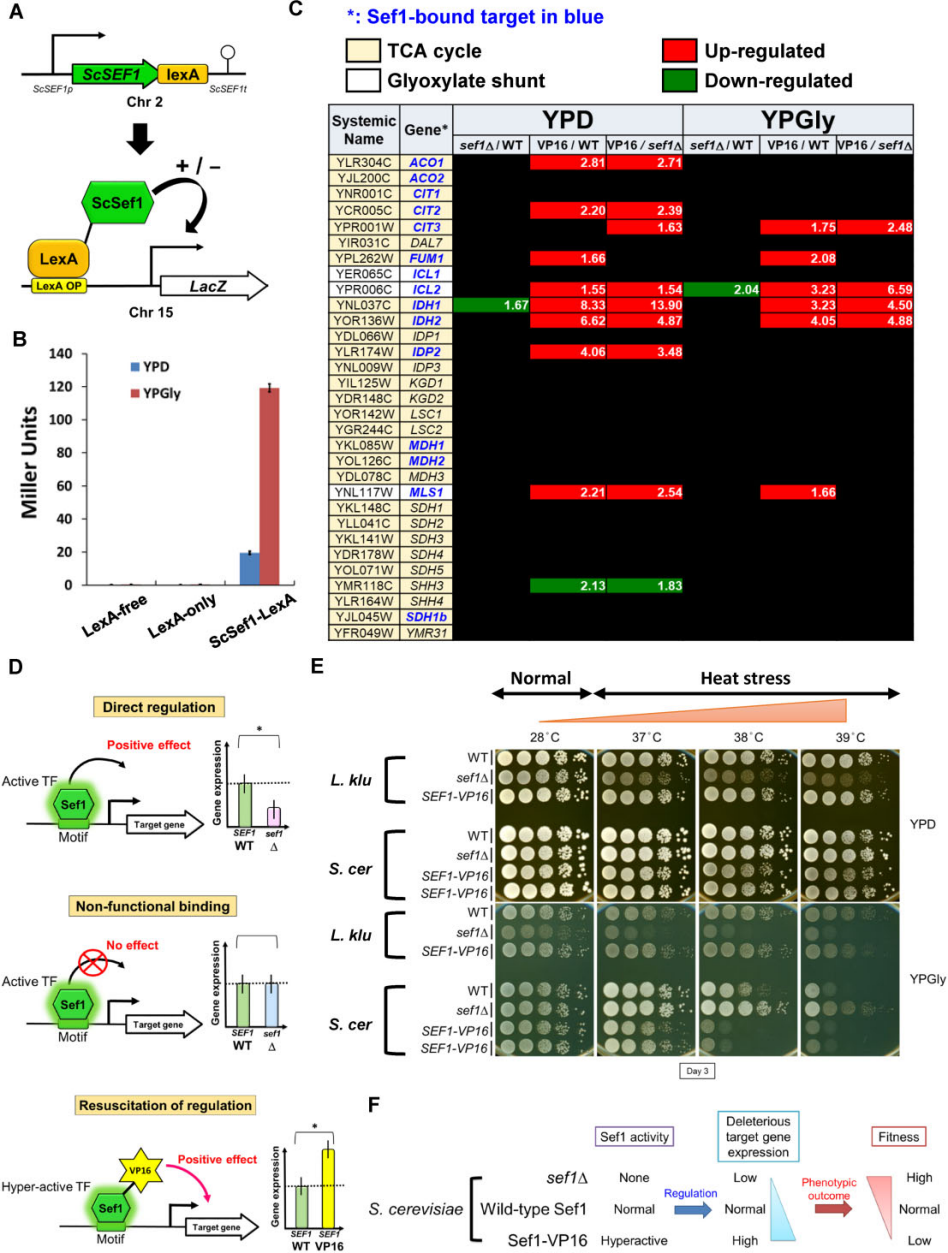
Sef1-mediated regulation of TCA cycle genes does not determine phenotypic outcomes in wild-type *S. cerevisiae*. (**A**) One-hybrid assays for *S. cerevisiae* Sef1. Native-promoter-driven ScSef1, which is C-terminally fused with the LexA DNA-binding domain, binds to the LexA operator (lexAOP) on the basal promoter of the lacZ reporter gene and modulates expression of the lacZ reporter. (**B**) Transcriptional activity of ScSef1, as measured by one-hybrid assays. ScSef1 activates transcription in response to both fermentative (glucose) and respiratory (glycerol) growth conditions. LacZ activity was measured using a liquid-galactosidase assay and results are displayed as mean Miller units ± standard deviation (SD) from three technical repeats. The LexA-only strain which showed a very low background level of LacZ activity similar to the non-tagging (LexA-free) strain was used as a negative control. The LexA DNA-binding domain alone had no artificial effect on transcription. (**C**) Differential expression of 28 TCA cycle and 3 glyoxylate shunt genes in response to *sef1*Δ and hyperactive *SEF1-VP16*. The RNA expression profiles from the *S. cerevisiae* wild-type, *sef1*Δ and *SEF1-VP16* (abbreviated as VP16 on the table) cells grown to early log-phase under both YPD and YPGly conditions were compared. The differential expression cut-off is ≥1.5-fold change, and the fold-change of each differentially expressed gene is indicated. A total of 12 TCA cycle genes and all 3 glyoxylate shunt genes are direct binding targets of Sef1 (based on ChIP-seq data). *sef1*Δ alone abolished expression of two genes, whereas hyperactive *SEF1-VP16* consistently resuscitated expression of more genes relative to either the wild-type or *sef1*Δ strains. (**D**) Three regulatory impacts of ScSef1 on its target genes. ‘Direct regulation’: Sef1 acts as a positive regulator, deletion of which downregulates gene expression. ‘Non-functional (TF) binding’: Sef1 is not required for expression of its binding targets, possibly due to redundancy (multiple redundant transcriptional regulators operate on the same promoter, with loss of any one of them potentially not altering final promoter activity) (23,84). This status results in comparable gene expression between the wild-type and *sef1*Δ strains. ‘Resuscitation of regulation’: By enhancing Sef1 transcriptional activity, non-functional Sef1 binding can partially and selectively (i.e. conditionally) become functional, allowing Sef1 to gain control of some target genes originally unaffected by it (23). This status is exemplified by the hyperactive *SEF1-VP16* mutant, which upregulates previously non-functionally bound target genes. (**E**) Growth of the *sef1*Δ and hyperactive *SEF1-VP16* mutants in response to YPD, YPGly and heat stress in both *L. kluyveri* and *S. cerevisiae*. All plates were incubated for 3 days. ScSef1 and LkSef1 displayed opposing effects on phenotypes. Two independent clones were tested for *S. cerevisiae SEF1-VP16* mutants. (**F**) Hypothetical model by which ScSef1 drives expression of species-specific deleterious target genes, with those genes inducing reduced fitness upon Sef1 activity increasing.

To examine the contribution of ScSef1 to gene expression, we adopted one-hybrid assays to assess ScSef1 activity (Figure 2A), which revealed that ScSef1 behaved like a transcriptional activator to induce the downstream LacZ reporter under fermentative conditions. Moreover, ScSef1 activity was dramatically enhanced when the yeast cells were grown in the respiratory medium (Figure 2B). Notably, *SEF1* does not have an ohnolog in *S. cerevisiae* and, unexpectedly, *SEF1* deletion downregulated only one TCA cycle gene (Figure 2C and Supplementary Tables S2 and S3), indicating that Sef1 does not exert direct regulation on the TCA cycle regulon (as depicted in Figure 2D, upper panel). Instead, wild-type Sef1 bound non-functionally to almost all (11 out of 12) ChIP targets of TCA cycle genes (Figure 2C and D, middle panel). Moreover, positive regulation could be resuscitated by deploying the hyperactive Sef1-VP16 mutant in which Sef1 was fused C-terminally to the activation domain of the VP16 viral protein known to interact with TBP (TATA-binding protein), TFIIB (Transcription factor II B) and the SAGA (Spt-Ada-Gcn5-acetyltransferase) histone acetylase complex to enhance yeast transcription (23,41) (Figure 2C and D, bottom panel). These findings demonstrate that although Sef1 can bind to the promoters of TCA cycle genes, the activation dose elicited by Sef1 binding is insufficient to drive expression of the majority of those targets.

In comparison to Sef1 binding-mediated regulation of TCA cycle genes in *C. albicans* (non-functional) (25) and *L. kluyveri* (moderate) (23), wild-type ScSef1 exerts a very weak regulatory role in the expression of TCA cycle genes (Supplementary Figure S2C). Unlike LkSef1 that is required for respiratory growth and heat stress tolerance in *L. kluyveri* (23), deletion of *SEF1* in *S. cerevisiae* did not cause growth defects (Figure 2E). In contrast, the *S. cerevisiae sef1*Δ mutant displayed enhanced fitness under heat-stressed respiratory conditions compared to the wild type, and the *SEF1-VP16* mutant displayed more pronounced growth defects relative to the wild type (Figure 2E). These results imply that ScSef1-mediated positive control of condition-specific deleterious gene(s) determines phenotypic outcomes (Figure 2F). In other words, we hypothesized that ScSef1 induces the expression of some deleterious genes which exert stronger phenotypic effects under respiratory and heat-stressed conditions. Moreover, these deleterious target genes are most likely different from the TCA cycle genes.

### *NDE1* is the major phenotype-determining gene regulated by ScSef1

**Figure 3.**
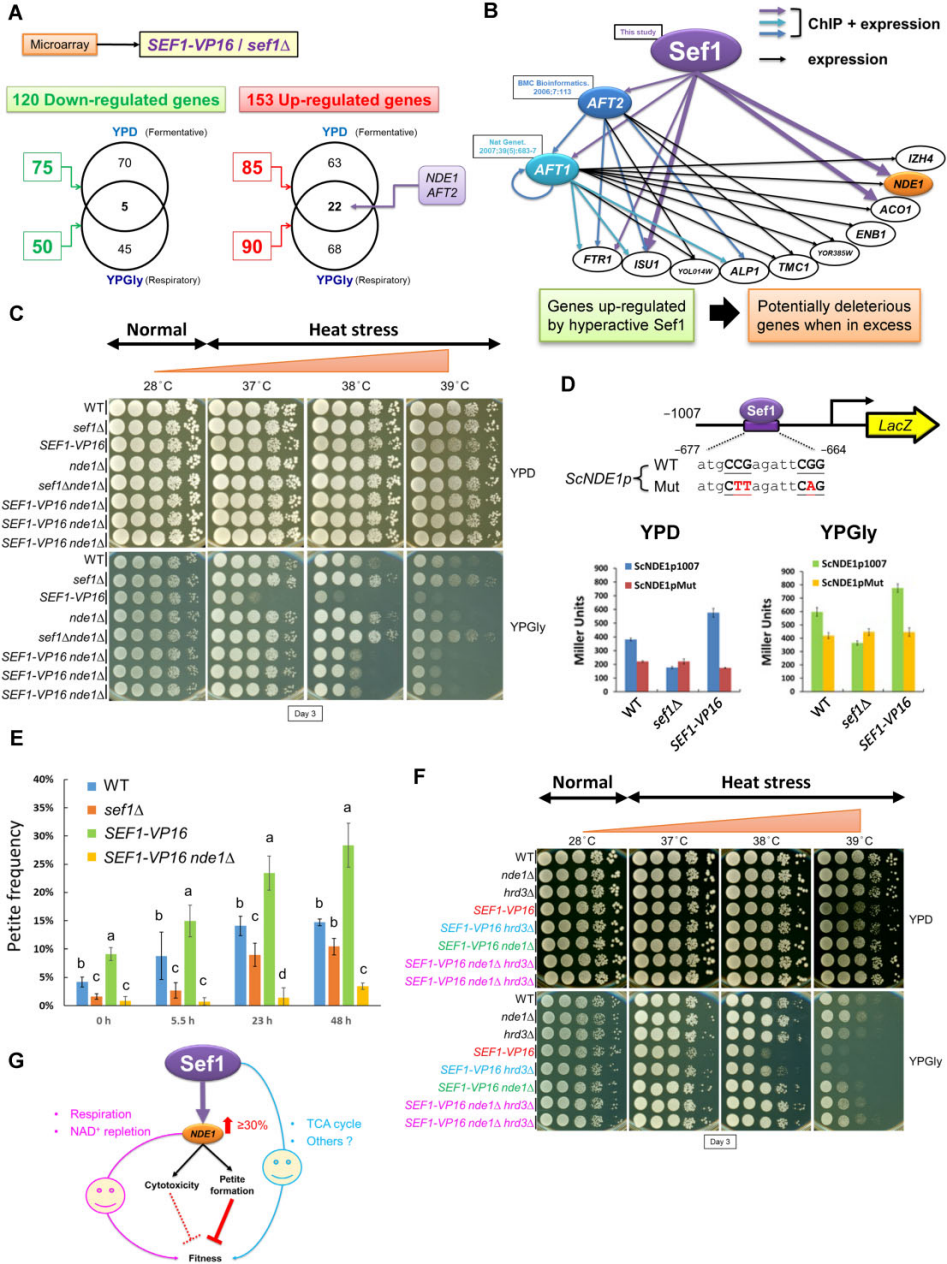
*NDE1* is the phenotype-determining gene of the Sef1-mediated transcription regulatory network in *S. cerevisiae*. (**A**) Summarized total numbers of differentially expressed genes in response to the hyperactive *SEF1-VP16* versus the *sef1*Δ mutant under YPD and YPGly conditions. There are 120 downregulated and 153 up-egulated genes under either condition. Notably, *NDE1* and *AFT2* are upregulated under both conditions. (**B**) The Sef1-Aft2 subnetwork is composed of three TFs (including Sef1, Aft2 and Aft1) and ten *SEF1-VP16* upregulated genes (Supplementary Tables S4–S7), which are also either Aft2 or Aft1 target genes and have been reported as deleterious at higher abundance (from Saccharomyces Genome Database phenotypes). The edges (expression and ChIP-binding connections) of this network are built according to data from this study (Supplementary Table S1) and previous reports (85,86). *NDE1* is highlighted in orange. (**C**) Growth of *SEF1-VP16 nde1*Δ mutants in response to YPD, YPGly and heat stress in *S. cerevisiae*. All plates were incubated for 3 days. Three independent *SEF1-VP16 nde1*Δ clones were tested. Deletion of *NDE1* can considerably rescue the growth defects generated by hyperactive Sef1. (**D**) Design of the verification system for Sef1 binding on the *NDE1* promoter by the LacZ reporter-based promoter assay. Two consensus Gs and one consensus C were mutated to A or T (highlighted in red) (upper panel). The ChIP peak regions for the *S. cerevisiae NDE1* promoters (−1 to −1007 from ATG) contain only one high-confidence (*P*-value <0.0001) Sef1 binding motif (Supplementary Figure S1D). The wild-type and motif-mutated promoters were fused to the LacZ reporter gene. The contribution of Sef1 motifs to promoter activities was assayed in wild-type, *sef1*Δ and hyperactive *SEF1-V16* cells (bottom panel). LacZ activity was measured under both YPD and YPGly conditions using a liquid-galactosidase assay. The β-galactosidase levels are displayed as average Miller units ± SD from three technical repeats. Compared to the wild-type strain with the wild-type *NDE1* promoter, Sef1 binding contributes to ∼40% and ∼30% of promoter activities under YPD and YPGly conditions, respectively. Moreover, the enhanced Sef1 activity from VP16 contributes to ∼50% and ∼30% of additional promoter activities under YPD and YPGly conditions, respectively. (**E**) The petite-inducing effects of Sef1 and Nde1. Petite formation was induced by growth in YPD at 39°C. The petite cells were counted at the indicated time points, and petite frequency is displayed as mean ± SD from five technical repeats. One-way ANOVA followed by Tukey’s multiple comparisons post hoc test was performed, with letters indicating significant differences. Petite formation rates increased with increasing Sef1 activity (*sef1*Δ, wild type, to *SEF1-VP16*) and required the presence of Nde1 (*SEF1-VP16 nde1*Δ). (**F**) Growth of the *SEF1-VP16 hrd3*Δ mutants in response to YPD, YPGly and heat stress in *S. cerevisiae*. All plates were incubated for 3 days. Two independent *SEF1-VP16 nde1*Δ*hrd3*Δ clones were tested. Abolishing the Hrd3 function enhances the stability of mitochondrial DNA (mtDNA), thereby reducing the petite formation rate (45). Deletion of *HRD3* can partially rescue the defective growth caused by Sef1 hyperactivity, despite this rescue effect being weaker than the effect of *nde1*Δ. There is no clear additive rescue effect when both *HRD3* and *NDE1* are deleted. (**G**) A working model depicting that ScSef1-mediated *NDE1* expression determines cellular fitness via multiple moonlighting mechanisms, including two beneficial functions in respiration and NAD^+^ repletion and two deleterious functions in cytotoxicity (only when ultra-highly expressed) (44) and petite formation. Modulating the activities of Sef1 generally affects fitness by changing Nde1-driven petite formation rates in the population. In the presence of Nde1, the potentially positive effects of the other Sef1-regulated genes on fitness are much less significant.

To identify candidate phenotype-determining genes in the ScSef1 regulatory network, we focused on the most differentially expressed genes (DEGs) in response to Sef1 activity by comparing the transcriptional profiles between the *SEF1-VP16* and *sef1*Δ strains (Figure 3A), resulting in the selection of six candidates from 153 upregulated genes (Supplementary Figure S3A). There were five selection criteria for candidates: (1) the candidate genes were upregulated in response to the hyperactive Sef1 under both YPD and YPGly conditions, (2) deleterious when overexpressed or (3) beneficial when deleted, (4) ScSef1 binding target genes and (5) not essential for respiratory growth so that they can be deleted to assess the rescue effect. Moreover, these 153 genes display GO terms related to iron homeostasis, pre-ribosome biogenesis, TCA cycle and peroxisome, and sugar transport based on STRING analysis (Supplementary Figure S3B), with the promoters of the six candidate DEGs with previously established deleterious effects (from SGD ‘Phenotypes’ annotations) being bound by Sef1. Intriguingly, before this study, there were no notable genetic or physical interactions among these six candidate targets and Sef1 (Supplementary Figure S3C), implying that they may affect phenotypes independently rather than cooperatively. Consequently, we chose for verification the most highly abundant phenotype-determining gene candidate, *NDE1* (Supplementary Figure S3A), and another candidate, *AFT2*, representing a Sef1-targeted transcriptional regulator potentially responsible for secondary expression of phenotype-determinant genes (Supplementary Figure S4A and B). We also generated a Sef1-Aft2 subnetwork to represent the potential regulatory cascade involved in transcription of these phenotype-determinant genes (Figure 3B). As expected, a *nde1*Δ mutant significantly rescued the growth defects elicited by Sef1 hyperactivity (Figure 3C), whereas the *aft2*Δ mutant displayed only slightly enhanced fitness, especially at 37°C (Supplementary Figure S4C). Moreover, double deletion of *NDE1* and *AFT2* did not result in a clear additive beneficial effect (Supplementary Figure S4D), indicating that Sef1 modulates fitness more directly via Nde1 activity than indirectly through Aft2.

To further scrutinize how Sef1 regulates *NDE1*, we mutated the unique high-confidence Sef1 motif on the *NDE1* promoter (Supplementary Figure S1D) to potentially interfere with Sef1 binding (Supplementary Figures S5A–E) and thus to compromise Sef1-contributed promoter activity (Figure 3D, upper panel). Similar to the *sef1*Δ mutant, mutating this motif reduced by ∼40% and ∼30% *NDE1* promoter activities relative to that of wild type under YPD and YPGly conditions, respectively (Figure 3D, bottom panel), thereby confirming that ScSef1 directly and positively regulates *NDE1*.

### Moonlighting functions of Nde1 in *S. cerevisiae* petite cell formation.

ScNde1 is a pleiotropic protein that not only acts as a NADH dehydrogenase required for respiration and NAD + replenishment (42,43), but also exerts seemingly deleterious roles by inducing cell death (cytotoxicity) of respiration-compromised cells (44) and by promoting basal and stress-induced petite formation (45). The deleterious functions of ScNde1 are independent of its NADH dehydrogenase activity in respiration (44), supporting that ScNde1 is a moonlighting protein that exerts multiple functions via different mechanisms. To establish how ScSef1 activity regulates fitness via Nde1, we first mimicked hyperactive Sef1-induced *NDE1* expression by ectopically expressing an additional copy of *NDE1* and tested the cytotoxic effects of *NDE1* overexpression on respiration-compromised cells by using yeast petite strains. Interestingly, overexpressed ScNde1 only induced very weak cytotoxicity in petite cells (Supplementary Figure S6A). Moreover, enhanced *SEF1-VP16*-mediated overexpression did not exaggerate the cytotoxic effects (Supplementary Figure S6B). Together, these results indicate that a mildly elevated Nde1 level (either via hyperactive Sef1 or centromeric plasmid-based ectopic expression) is insufficient to trigger severe cytotoxicity, in contrast to the case of ultra-high overexpression by the *GAL* promoter (44). Taken together, this evidence indicates that cytotoxicity from elevated levels of Nde1 is not the major mechanism by which Sef1-mediated deleterious phenotypes are triggered.

The defective mitochondrial functions make yeast petite cells not only metabolically sick due to an insufficiency in the biosynthesis of important amino acids, distorted iron metabolism and an inhibited TCA cycle (46), but also more sensitive to environmental perturbations, especially heat stress (47), possibly due to the depletion of adenosine triphosphate (ATP) demands (29) required for the heat-shock response (48) and impaired mitochondrion-assisted protein quality control systems required for reducing heat stress-induced proteotoxicity (49,50). Therefore, we hypothesized that fitness is determined by the frequency of the less-fit petite cells in the population. To test that possibility, we quantified petite formation rates under heat-stressed conditions (Figure 3E). Relative to wild-type cells, petite frequency was lower for the *sef1*Δ cells and higher for cells expressing hyperactive Sef1. Moreover, this increase in petite frequency in the *SEF1-VP16* strain depended on Nde1 since deletion of *NDE1* attenuated the petite-eliciting effect of hyperactive Sef1 (Figure 3E). Thus, basal and heat-stress-induced petite frequencies are positively correlated with Sef1 activity and consequent Nde1 levels (Figure 3D). To corroborate this evidence, we tested if other mtDNA-stabilizing mutants could rescue the fitness defect of *SEF1-VP16* strains. Hrd3 is a component of the HMG-CoA reductase degradation (HRD) complex involved in the ER (endoplasmic reticulum)-associated degradation pathway (51), and it has been suggested to negatively regulate mtDNA stability (45). *HRD3* loss-of-function has been proven to reduce yeast petite formation rates (45). Bre5 is a ubiquitin protease cofactor that forms a deubiquitination complex with Ubp3 (52). An evolved yeast strain with enhanced mtDNA stability was found to possess a *BRE5* missense mutation (most likely a loss-of-function mutation) (45), with this phenotype potentially attributable to derepressed mitophagy arising from the *bre5* mutation (53). As expected, we found that enhancing mtDNA stability (i.e. reducing petite frequency) by deleting *HRD1* or *BRE5* partially rescued the growth defects caused by *SEF1-VP16* expression (Figure 3F and Supplementary Figure S6C). Notably, double deletion of *HRD3* and *NDE1* did not exert an additive effect relative to *nde1*Δ, implying that *NDE1* is epistatic to *HRD3* in how mtDNA stability is regulated.

Glutathione (γ-glutamylcysteinylglycine; GSH) is a low molecular-mass thiol that participates in many cellular processes in yeast (54), including the maintenance of mitochondrial functions (55,56). To confirm that petite cells are the major determinant of Sef1-mediated population fitness, we first demonstrated that GSH supplementation did not ameliorate the elevated petite formation triggered by a high level of Nde1 (Supplementary Figure S7A), and then demonstrated that additional GSH directly mitigated the growth defect of *NDE1*-overexpressing petite cells under heat stress (Supplementary Figure S7B). Consistently, repletion of GSH or its precursor enhanced the growth of Sef1-hyperactive cells but did not generate comparable effects on the wild-type population containing a low frequency of petite cells (Supplementary Figure S8A and B). Taken together, we proved that increasing the fitness of slow-growing petite individuals in the hyperactive Sef1 population by GSH supplementation can enhance its population growth rate. These results indicate that ScSef1 regulates population fitness primarily via Nde1-mediated petite formation, at least under the conditions we tested (Figure 3G), showing that hyperactive Sef1 population fitness can be increased by either reducing the petite formation (Figure 3E and F, and Supplementary Figure S6C) or by directly enhancing the fitness of the petite cells (Supplementary Figure S8A and B).

### Preservation of the conditionally deleterious Sef1-*NDE1* pathway in *S. cerevisiae*

**Figure 4.**
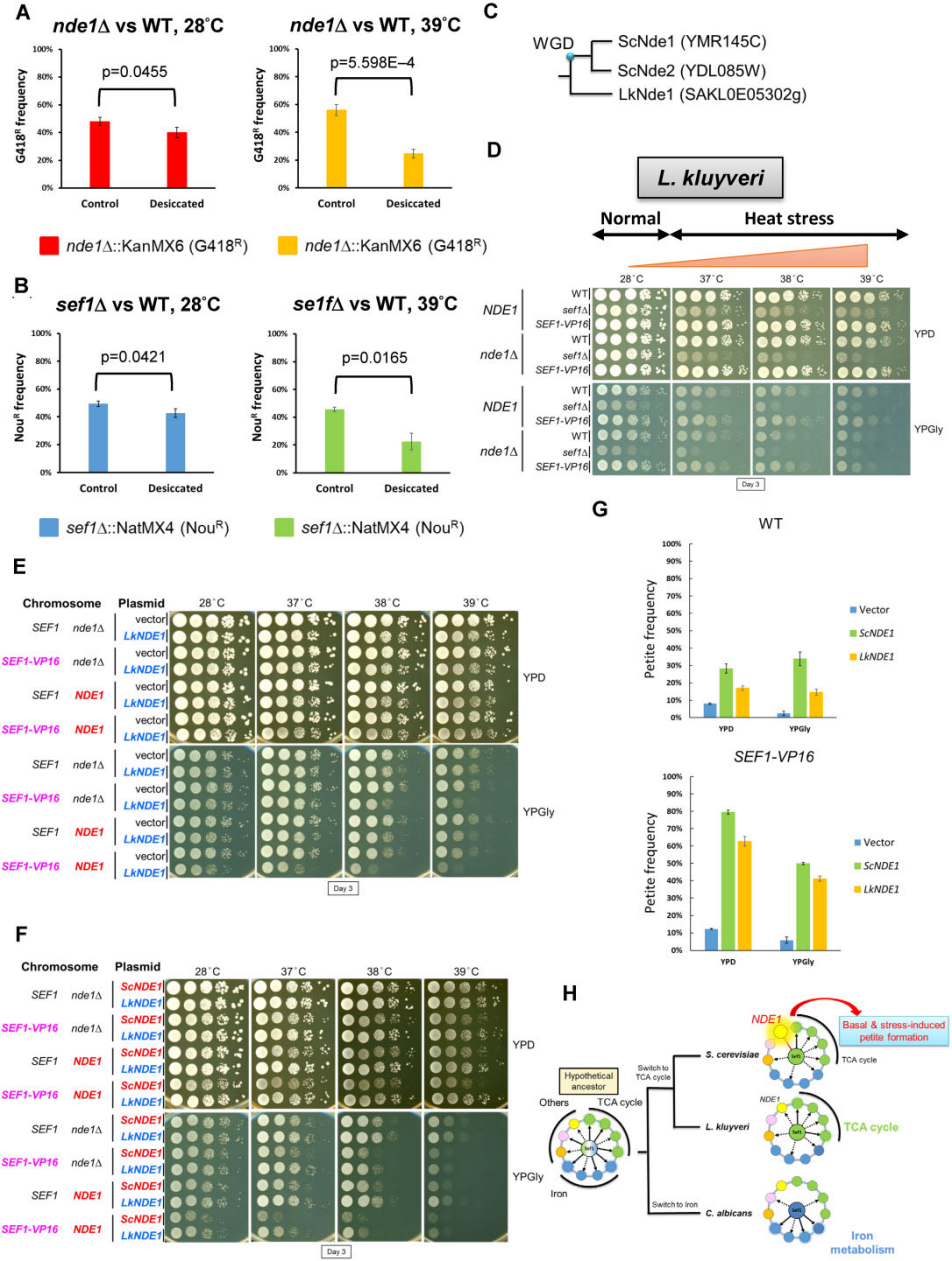
*Saccharomyces cerevisiae* is more vulnerable to the petite-inducing effect of Nde1. (**A**) Nde1 contributes to desiccation tolerance. (**B**) Sef1 contributes to desiccation tolerance. For (**A**) and (**B**), desiccation assays were performed by mixing the drug-resistant deletion strains with the reference drug-sensitive wild-type strain at an initial 50:50 cell number ratio and air-dried for 24 h at 28 or 39°C. The frequency of the drug-resistant cells is shown. A two-tailed Student’s *t*-test was used to calculate the *P*-value for each pair by comparing the drug-resistant frequency of the control sample (without desiccation) with the desiccated sample. (**C**) Orthology of Nde1 between *S. cerevisiae* and *L. kluyveri*. There is only one Nde1 homolog in *L. kluyveri*. LkSef1 also binds to the *NDE1* promoter (23). (**D**) Growth of the *SEF1-VP16 nde1*Δ mutants in response to YPD, YPGly and heat stress in *L. kluyveri*. All plates were incubated for 3 days. Deletion of *NDE1* has no clear effect on cell growth, even under respiratory conditions. (**E**) Ectopic expression of the *LkNDE1* homolog on a centromeric plasmid selected by HGB in *S. cerevisiae*. Relative to vector-only controls, additional expression of *LkNDE1* exacerbates the growth defect, especially in strains harboring *SEF1-VP16* and/or a chromosome copy of *ScNDE1*. (**F**) Ectopic *LkNDE1* is less deleterious than *ScNDE1* when additionally expressed in *S. cerevisiae*. For (**E**) and (**F**), cells were spotted and grown in YPD or YPGly, and with/without heat stress in the presence of 300 μg/ml HGB. All plates were incubated for 3 days. (**G**) Basal petite-inducing effects in response to ectopic expression of *LkNDE1* and *ScNDE1*. Petite formation was induced by growth in YPD + HGB at 28°C for 20 h. Petite cells were counted by spreading them on YPD plates. Petite frequency is displayed as mean ± SD from five technical repeats. Relative to ectopic *ScNDE1*, ectopic *LkNDE1* is less petite-inducing in both the wild-type and *SEF1-VP16* backgrounds. (**H**) Proposed switches in Sef1 function from a hypothetical common ancestor to the non-regulating branch (*C. albicans*) and TCA cycle-regulating branch (23). The divergence between *L. kluyveri* and *S. cerevisiae* leads to a split of the true-TCA cycle regulating sub-branch and the pseudo-TCA cycle regulating sub-branch, with the phenotypic outcomes of this latter being dominated by the petite-inducing activity of the conserved Sef1 target gene, *NDE1*.

The existence of the strong deleterious moonlighting function of Nde1 raises the question as to why *S. cerevisiae* has preserved the Sef1-*NDE1* pathway. Many genes involved in respiration were shown previously to contribute to desiccation resistance (57). Therefore, we evaluated the essentiality of Sef1 and Nde1 under conditions of desiccation, representing dehydration stress. From survival assays, the *nde1*Δ and *sef1*Δ mutants showed lower relative fitness (survival rate) than wild type in response to desiccation, especially under concomitant heat-stressed conditions (Figure 4A and B). Next, we compared the viability of *S. cerevisiae* strains exhibiting different levels of petite formation in continuous respiratory cultures. We observed that viability decreased according to the petite formation rate driven by reduced *NDE1* activity (Supplementary Figure S9A). Furthermore, we manipulated *NDE1* expression levels by changing the Sef1-binding motif on the *NDE1* native promoter (Figure 3D) and surprisingly observed that intermediate *NDE1* activity proved the most deleterious, i.e. elicited the lowest viability in continuous respiratory culture (Supplementary Figure S9B). This outcome indicates that Nde1 represents a ‘dilemma protein’, on the one hand endowing beneficial effects in terms of respiratory growth, but on the other hand, having deleterious impacts through petite formation, and it implies that natural selection conditionally favors the preservation of the Sef1-*NDE1* pathway in *S. cerevisiae*.

### Relatively higher vulnerability of *S. cerevisiae* to the petite-inducing effect of Nde1 orthologs

Notably, *L. kluyveri* only has one Nde1 ortholog (Figure 4C) and it is not deleterious in this species under the *SEF1-VP16* or *sef1*Δ genetic backgrounds (Figure 4D). However, ectopic expression of *L. kluyveri NDE1* in *S. cerevisiae* not only induced mild growth defects when expressed alone, but also exacerbated the growth defects caused by different expression levels of *S. cerevisiae NDE1* (Figure 4E). Interestingly, LkNde1 is less deleterious than ScNde1 when expressed in *S. cerevisiae* under the same genetic background (Figure 4F). Commensurately, LkNde1 is also petite-inducing when expressed in *S. cerevisiae*, but less so than ScNde1 (Figure 4G).

Taken together, we propose an evolutionary trajectory model (Figure 4H) by which the Sef1 TRN evolved and diverged into an iron-regulating branch (*C. albicans*) and a TCA cycle-regulating branch, with this latter subsequently diverging into a true-regulating sub-branch (*L. kluyveri*) and a pseudo-regulating sub-branch (*S. cerevisiae*). In pseudo-TCA cycle-regulating species, Sef1-mediated regulation of TCA cycle genes does not determine phenotypic outcomes. However, the phenotype (i.e. fitness) is prompted by the petite-inducing effect of the Sef1 target gene, Nde1, the orthologs of which are conserved targets of Sef1 in both *S. cerevisiae* and *L. kluyveri*. In conclusion, we define this particular evolutionary trajectory as ‘phenotypic masking’ in which a target gene exerting a strong phenotypic impact masks the phenotypic outputs of other target genes in a TRN (Supplementary Figure S10A), whereby the differing phenotypic effects of a target gene conserved between two generally comparable TRNs lead to ‘deceptive’ disruptive rewiring, i.e. although disruptive rewiring is still underway, the TRN has already displayed a different phenotype seemingly (but not really) resulting from a complete regulatory dissociation between the TF and its original phenotype-determining genes.

## Discussion

### Possible causes of evolutionary divergence in orthologous Sef1 TRNs across yeasts

Sef1 is a relatively young gene and it appears to have arisen during the divergence between the *Pezizomycotina* and *Saccharomycotina* clades (Figure 1F). Our previous preliminary study comparing the phenotypes of *sef1*Δ mutants with each respective wild type of different yeasts indicated that Sef1 orthologs regulate iron-dependent, respiration-dependent, heat stress-dependent and potentially other species-specific functions (23). Theoretically, such functional divergence of TRNs is a consequence of disruptive rewiring. However, in this study, we have demonstrated that deletion of two Sef1 orthologs leads to opposite phenotypic impacts, i.e. a transition alternating between beneficial and deleterious outcomes (Figure 2E). This transition may not only require species-specific changes in the protein activities of conserved Sef1 target genes, such as *NDE1* (Figures 3C and 4D), but also changes in species-specific genomic backgrounds (Figure 4E and F). Here, we discuss four factors that potentially contribute to how Sef1 TRNs have evolved. First, the regulatory dissociation between LkSef1 and Lk*NDE1* [i.e. deletion of *SEF1* did not downregulate the expression of *NDE1* in *L. kluyveri* despite that LkSef1 binds strongly to the promoter of Lk*NDE1* (23)] may directly contribute to this phenotypic divergence between the Sef1 TRNs in *S. cerevisiae* and *L. kluyveri*. Second, independent development of the ability to tolerate mtDNA loss among many yeast species (also called ‘petite-positive’ species, such as those in the *Saccharomyces* clade) (58) has endowed *S. cerevisiae* populations with the ability to maintain viable but less-fit petite cells. Third, evolutionary loss of classical mitochondrial respiratory Complex I genes in the *Saccharomycetaceae* family (59) and its subsequent evolution of functional compensation via alternative NADH dehydrogenases (42,60) allows the yeast cells to bypass Complex I electron transport by oxidizing NADH without proton pumping (61), possibly further promoting the evolution of alternative NADH dehydrogenases via duplications (62) and entrenchment of Nde1-related moonlighting functions (44,45). Finally, by evolving different capabilities of anaerobic growth and preferences for a fermentative lifestyle (i.e. Crabtree effect) (58,63,64), yeasts may also relax selection pressures and enable innovative Nde1 moonlighting functions (65–67).

Moreover, by comparing the yeast species of the iron-regulating branch (i.e. *C. albicans*) with the species of the TCA cycle-regulating branch (i.e. *L. kluyveri* and *S. cerevisiae*) (Figure 4H), we also notice some potential connections between the habitats of these yeasts and their Sef1 functions. As a Crabtree-negative (68) and opportunistic pathogen (69), *C. albicans* cells utilize glucose more stably and efficiently from the host and simultaneously maintain the metabolic plasticity for respiratory growth [reviewed in (70)] without repressing the stress resistance which is vital for *C. albicans* survival in response to the host immune system (71). Hypothetically, *C. albicans* does not need its Sef1 to fine-tune the respiration-related genes, such as TCA cycle genes, and consequently evolved to use Sef1 to regulate iron-uptake, which is crucial for its pathogenesis [reviewed in (70)]. In contrast, possibly due to the variable glucose availability in natural environments, the weak Crabtree-positive yeast *L. kluyveri* and the Crabtree-positive yeast *S. cerevisiae* (72) which have a clear metabolic diauxic shift, still maintain the Sef1-mediated regulation of respiration, including TCA cycle and Nde1 in the electron transport chain.

### Putative mechanisms of Nde1’s petite-inducing activity

It remains unclear why Nde1 abundance is correlated with mtDNA instability. One hypothesis is that Nde1-induced excess electron leakage and consequent ROS (reactive oxygen species) formation in mitochondria directly damages mtDNA (73). However, in some cases, ROS levels are not correlated with petite formation rates (73,74) or ATP energy production (75), raising the possibility that excess ROS is simply a consequence of the perturbed electron transport chain and not the cause of mtDNA instability. Therefore, we propose an alternative hypothesis.

Petite formation is the result of insufficient selection against mutant mtDNA. In yeast, proper selection of functional mtDNA in a heteroplasmic mitochondrial network relies on normal biogenesis and intact morphology of cristae (76), which generate cristae subdomains composed of functional mtDNA and the proteins (respiratory chain supercomplex) it encodes. These domains create a spatial ‘sphere of influence’ that hypothetically differentiates mutant and functional mtDNA. The relationship between cristae and mtDNA has been explored extensively in yeast and mammals (77,78). Notably, mitochondrial protein import plays an important role in respiratory chain supercomplex assembly (79), with import fluency being required for cell growth under respiratory conditions (50). Tom70 is a key component of the TOM (translocase of outer membrane) complex involved in recognizing and importing all nascent mitochondrial proteins (80). Moreover, Tom70 moonlights to regulate the transcriptional activity of mitochondrial proteins, with its expression levels being correlated with mtDNA abundance (81). Furthermore, Tom70 and MitoStore protein deposits (possibly independently) recruit cytosolic chaperones to trap mitochondrial precursor proteins and/or cytosolic protein aggregates (49,50,82). These organelle-condensate interactions potentially provide a cellular reservoir to reduce proteotoxicity and to recycle proteins after proteostatic or mitochondrial stress has alleviated.

Accordingly, we postulate that Nde1 affects mtDNA quality control by acting as a rate-limiting cargo to determine the smoothness of mitochondrial protein import. An inappropriate Nde1 level (e.g. via overexpression) may globally overwhelm and clog the mitochondrial protein import machinery, thereby disrupting cristae biogenesis and maintenance, and ultimately affecting mtDNA stability. However, this hypothesis remains to be tested, warranting further experiments.

### Flexible Nde1 expression controls the phenotypic plasticity of the Sef1 TRN in *S. cerevisiae*

Our study has demonstrated that Nde1 is deleterious under heat-stressed conditions (Figure 3C), but it can still be beneficial under desiccated conditions (Figure 4A), indicating that its deleterious petite-inducing activity can also be advantageous under a different environment (Supplementary Figure S9A). Moreover, removing Sef1-elicited *NDE1* expression alone is more deleterious than complete deletion of *NDE1* itself (Supplementary Figure S9B). These results not only indicate that the fitness effects of Nde1 in *S. cerevisiae* result from a dynamic balance between its beneficial and deleterious activities in response to changing environments, but also imply that the ‘flexibility’ of *NDE1* expression is indeed the critical fitness determinant.

Commensurate with this notion of expression flexibility, *NDE1* expression is affected by deletion of at least 71 transcriptional or signaling factors according to 1484 deletome data (83), including important components of the retrograde response, Ras-cAMP-PKA signaling, glucose repression signaling, DNA repair and nitrogen catabolite repression pathways. Together with the high turnover rate (i.e. short half-life) of Nde1 proteins (44), this evidence supports that Nde1 abundance is subjected to refined control both in terms of RNA and protein levels. Our study demonstrates that Sef1 is one of the major regulators of Nde1 (Figure 3B and D), so herein we have proposed a model whereby the future evolution of ScSef1, unlike LkSef1, may be more constrained due to the limited flexibility (i.e. low plasticity) of one of its target genes, *NDE1*, highjacking global phenotypic outcomes (Supplementary Figure S10B).

### Some limitations of this study

Due to the lack of appropriate ancestral and intermediate species or strains which allow us to do molecular comparisons in microscopic scales, we are unable to address why and how the Sef1 TRNs in these two species ‘have to’ evolve divergently in these ways (i.e. what is the driving force or mechanisms behind the changes?). In other words, so far we are unable to elucidate how a TRN evolved from one pattern to another. Moreover, our ChIP-seq and gene expression data were all based on the YPD (fermentative) and YPGly (respiratory) conditions. Although they are the commonest and most representative growth conditions for yeasts, we are unable to exclude the possibility of getting different true and non-functional binding target genes of Sef1 in the other untested conditions.

## Supplementary Material

gkae1147_Supplemental_Files

## Data Availability

The ChIP-seq data have been submitted to the Gene Expression Omnibus (GEO) website (http://www.ncbi.nlm.nih.gov/geo/) under accession number GSE262389. The processed ChIP-seq results are shown in Supplementary Table S1. The microarray data have been submitted to the GEO website under accession number GSE262043. The processed results are shown in Supplementary Tables S2–S7.

## References

[1] Wagner G.P. , Lynch V.J The gene regulatory logic of transcription factor evolution. Trends Ecol. Evol. 2008; 23:377–385. 18501470 10.1016/j.tree.2008.03.006

[2] Mack K.L. , Nachman M.W Gene regulation and speciation. Trends Genet. 2017; 33:68–80. 27914620 10.1016/j.tig.2016.11.003 PMC5182078

[3] Carroll S.B. Endless forms: the evolution of gene regulation and morphological diversity. Cell. 2000; 101:577–580. 10892643 10.1016/s0092-8674(00)80868-5

[4] Romero I.G. , Ruvinsky I., Gilad Y. Comparative studies of gene expression and the evolution of gene regulation. Nat. Rev. Genet. 2012; 13:505–516. 22705669 10.1038/nrg3229 PMC4034676

[5] Scannell D.R. , Wolfe K. Rewiring the transcriptional regulatory circuits of cells. Genome Biol. 2004; 5:206. 14759249 10.1186/gb-2004-5-2-206 PMC395740

[6] Dalal C.K. , Johnson A.D. How transcription circuits explore alternative architectures while maintaining overall circuit output. Genes Dev. 2017; 31:1397–1405. 28860157 10.1101/gad.303362.117 PMC5588923

[7] Li H. , Johnson A.D. Evolution of transcription networks — lessons from yeasts. Curr. Biol. 2010; 20:R746–R753. 20833319 10.1016/j.cub.2010.06.056 PMC3438143

[8] Perez J.C. , Groisman E.A. Evolution of transcriptional regulatory circuits in bacteria. Cell. 2009; 138:233–244. 19632175 10.1016/j.cell.2009.07.002 PMC2726713

[9] Wang L. , Wang F.-F., Qian W. Evolutionary rewiring and reprogramming of bacterial transcription regulation. J. Genet. Genom. 2011; 38:279–288. 10.1016/j.jgg.2011.06.001 21777852

[10] Tanay A. , Regev A., Shamir R. Conservation and evolvability in regulatory networks: the evolution of ribosomal regulation in yeast. Proc. Natl Acad. Sci. U.S.A. 2005; 102:7203–7208. 15883364 10.1073/pnas.0502521102 PMC1091753

[11] Tuch B.B. , Galgoczy D.J., Hernday A.D., Li H., Johnson A.D. The Evolution of Combinatorial Gene Regulation in Fungi. PLoS Biol. 2008; 6:e38. 18303948 10.1371/journal.pbio.0060038 PMC2253631

[12] Lavoie H. , Hogues H., Mallick J., Sellam A., Nantel A., Whiteway M. Evolutionary tinkering with conserved components of a transcriptional regulatory network. PLoS Biol. 2010; 8:e1000329. 20231876 10.1371/journal.pbio.1000329 PMC2834713

[13] Hogues H. , Lavoie H., Sellam A., Mangos M., Roemer T., Purisima E., Nantel A., Whiteway M. Transcription Factor Substitution during the Evolution of Fungal Ribosome Regulation. Mol. Cell. 2008; 29:552–562. 18342603 10.1016/j.molcel.2008.02.006 PMC3838363

[14] Mallick J. , Whiteway M. The evolutionary rewiring of the ribosomal protein transcription pathway modifies the interaction of transcription factor heteromer Ifh1-Fhl1 (interacts with forkhead 1-Forkhead-like 1) with the DNA-binding specificity element*. J. Biol. Chem. 2013; 288:17508–17519. 23625919 10.1074/jbc.M112.436683 PMC3682550

[15] Martchenko M. , Levitin A., Hogues H., Nantel A., Whiteway M. Transcriptional rewiring of fungal galactose-metabolism circuitry. Curr. Biol. 2007; 17:1007–1013. 17540568 10.1016/j.cub.2007.05.017 PMC3842258

[16] Askew C. , Sellam A., Epp E., Hogues H., Mullick A., Nantel A., Whiteway M. Transcriptional regulation of carbohydrate metabolism in the human pathogen *Candida albicans*. PLoS Pathog. 2009; 5:e1000612. 19816560 10.1371/journal.ppat.1000612 PMC2749448

[17] Dalal C.K. , Zuleta I.A., Mitchell K.F., Andes D.R., El-Samad H., Johnson A.D. Transcriptional rewiring over evolutionary timescales changes quantitative and qualitative properties of gene expression. eLife. 2016; 5:e18981. 27614020 10.7554/eLife.18981 PMC5067116

[18] Tebung W.A. , Choudhury B.I., Tebbji F., Morschhäuser J., Whiteway M. Rewiring of the Ppr1 zinc cluster transcription factor from purine catabolism to pyrimidine biogenesis in the *Saccharomycetaceae*. Curr. Biol. 2016; 26:1677–1687. 27321996 10.1016/j.cub.2016.04.064

[19] Tsong A.E. , Miller M.G., Raisner R.M., Johnson A.D Evolution of a combinatorial transcriptional circuit: a case study in yeasts. Cell. 2003; 115:389–399. 14622594 10.1016/s0092-8674(03)00885-7

[20] Baker C.R. , Booth L.N., Sorrells T.R., Johnson A.D. Protein modularity, cooperative binding, and hybrid regulatory states underlie transcriptional network diversification. Cell. 2012; 151:80–95. 23021217 10.1016/j.cell.2012.08.018 PMC3519278

[21] Britton C.S. , Sorrells T.R., Johnson A.D Protein-coding changes preceded *cis*-regulatory gains in a newly evolved transcription circuit. Science. 2020; 367:96–100. 31896718 10.1126/science.aax5217 PMC8284397

[22] Nocedal I. , Mancera E., Johnson A.D. Gene regulatory network plasticity predates a switch in function of a conserved transcription regulator. eLife. 2017; 6:e23250. 28327289 10.7554/eLife.23250 PMC5391208

[23] Hsu P.-C. , Lu T.-C., Hung P.-H., Jhou Y.-T., Amine A.A.A., Liao C.-W., Leu J.-Y. Plastic rewiring of Sef1 transcriptional networks and the potential of nonfunctional transcription factor binding in facilitating adaptive evolution. Mol. Biol. Evol. 2021; 38:4732–4747. 34175931 10.1093/molbev/msab192 PMC8557406

[24] Gerwien F. , Safyan A., Wisgott S., Hille F., Kaemmer P., Linde J., Brunke S., Kasper L., Hube B. A novel hybrid iron regulation network combines features from pathogenic and nonpathogenic yeasts. mBio. 2016; 7:e01782-16. 27795405 10.1128/mBio.01782-16 PMC5082906

[25] Chen C. , Pande K., French S.D., Tuch B.B., Noble S.M. An iron homeostasis regulatory circuit with reciprocal roles in *Candida albicans* commensalism and pathogenesis. Cell Host Microbe. 2011; 10:118–135. 21843869 10.1016/j.chom.2011.07.005 PMC3165008

[26] Hsu P.-C. , Yang C.-Y., Lan C.-Y *Candida albicans* Hap43 is a repressor induced under low-iron conditions and is essential for iron-responsive transcriptional regulation and virulence. Eukaryot. Cell. 2011; 10:207–225. 21131439 10.1128/EC.00158-10 PMC3067405

[27] Johnson A.D. The rewiring of transcription circuits in evolution. Curr. Opin. Genet. Dev. 2017; 47:121–127. 29120735 10.1016/j.gde.2017.09.004 PMC6901287

[28] Choudhury B.I. , Whiteway M. Evolutionary transition of GAL regulatory circuit from generalist to specialist function in ascomycetes. Trends Microbiol. 2018; 26:692–702. 29395731 10.1016/j.tim.2017.12.008

[29] Day M. Sariaslani S. , Gadd G. M. Advances in Applied Microbiology. 2013; Vol. 85:San Diego, CA, USA Academic Press 1–41.

[30] Xu L. , Dong Z., Fang L., Luo Y., Wei Z., Guo H., Zhang G., Gu Y.Q., Coleman-Derr D., Xia Q. et al. OrthoVenn2: a web server for whole-genome comparison and annotation of orthologous clusters across multiple species. Nucleic Acids Res. 2019; 47:W52–W58. 31053848 10.1093/nar/gkz333 PMC6602458

[31] Shevchuk N.A. , Bryksin A.V., Nusinovich Y.A., Cabello F.C., Sutherland M., Ladisch S. Construction of long DNA molecules using long PCR-based fusion of several fragments simultaneously. Nucleic Acids Res. 2004; 32:e19–e19. 14739232 10.1093/nar/gnh014 PMC373371

[32] Reuß O. , Vik Å., Kolter R., Morschhäuser J. The SAT1 flipper, an optimized tool for gene disruption in *Candida albicans*. Gene. 2004; 341:119–127. 15474295 10.1016/j.gene.2004.06.021

[33] Babaei M. , Sartori L., Karpukhin A., Abashkin D., Matrosova E., Borodina I. Expansion of EasyClone-MarkerFree toolkit for *Saccharomyces cerevisiae* genome with new integration sites. FEMS Yeast Res. 2021; 21:foab027. 33893795 10.1093/femsyr/foab027 PMC8112480

[34] Sambrook J. Molecular Cloning: A Laboratory Manual. 2001; Third edition Cold Spring Harbor, N.Y. Cold Spring Harbor Laboratory Press.

[35] Sharma K.G. , Kaur R., Bachhawat A.K. The glutathione-mediated detoxification pathway in yeast: an analysis using the red pigment that accumulates in certain adenine biosynthetic mutants of yeasts reveals the involvement of novel genes. Arch. Microbiol. 2003; 180:108–117. 12819858 10.1007/s00203-003-0566-z

[36] Lai-Zhang J. , Xiao Y., Mueller D.M Epistatic interactions of deletion mutants in the genes encoding the F1-ATPase in yeast Saccharomyces cerevisiae. EMBO J. 1999; 18:58–64. 9878050 10.1093/emboj/18.1.58 PMC1171102

[37] Hsu P.C. , Cheng Y.H., Liao C.W., Litan R.R.R., Jhou Y.T., Opoc F.J.G., Amine A.A.A., Leu J.Y Rapid evolutionary repair by secondary perturbation of a primary disrupted transcriptional network. EMBO Rep. 2023; 24:e56019. 37009824 10.15252/embr.202256019 PMC10240213

[38] Machanick P. , Bailey T.L. MEME-ChIP: motif analysis of large DNA datasets. Bioinformatics. 2011; 27:1696–1697. 21486936 10.1093/bioinformatics/btr189 PMC3106185

[39] Grant C.E. , Bailey T.L., Noble W.S. FIMO: scanning for occurrences of a given motif. Bioinformatics. 2011; 27:1017–1018. 21330290 10.1093/bioinformatics/btr064 PMC3065696

[40] Assaad H.I. , Zhou L., Carroll R.J., Wu G. Rapid publication-ready MS-Word tables for one-way ANOVA. Springer Plus. 2014; 3:474. 25191639 10.1186/2193-1801-3-474 PMC4153873

[41] Hall D.B. , Struhl K. The VP16 activation domain interacts with multiple transcriptional components as determined by protein–protein cross-linking *in vivo**. J. Biol. Chem. 2002; 277:46043–46050. 12297514 10.1074/jbc.M208911200

[42] Luttik M.A.H. , Overkamp K.M., Kötter P., de Vries S., van Dijken J.P., Pronk J.T. The *Saccharomyce**s cerevisiae* NDE1 and NDE2 Genes Encode Separate Mitochondrial NADH dehydrogenases catalyzing the oxidation of cytosolic NADH*. J. Biol. Chem. 1998; 273:24529–24534. 9733747 10.1074/jbc.273.38.24529

[43] Croft T. , Venkatakrishnan P., Lin S.-J. NAD+ metabolism and regulation: lessons from yeast. Biomolecules. 2020; 10:330. 32092906 10.3390/biom10020330 PMC7072712

[44] Saladi S. , Boos F., Poglitsch M., Meyer H., Sommer F., Mühlhaus T., Schroda M., Schuldiner M., Madeo F., Herrmann J.M. The NADH dehydrogenase Nde1 executes cell death after integrating signals from metabolism and proteostasis on the mitochondrial surface. Mol. Cell. 2020; 77:189–202. 31668496 10.1016/j.molcel.2019.09.027

[45] Amine A.A.A. , Liao C.-W., Hsu P.-C., Opoc F.J.G., Leu J.-Y. Experimental evolution improves mitochondrial genome quality control in *Saccharomyces cerevisiae* and extends its replicative lifespan. Curr. Biol. 2021; 31:3663–3670. 34192514 10.1016/j.cub.2021.06.026

[46] Vowinckel J. , Hartl J., Marx H., Kerick M., Runggatscher K., Keller M.A., Mülleder M., Day J., Weber M., Rinnerthaler M. et al. The metabolic growth limitations of petite cells lacking the mitochondrial genome. Nat. Metab. 2021; 3:1521–1535. 34799698 10.1038/s42255-021-00477-6 PMC7612105

[47] Zubko E.I. , Zubko M.K. Deficiencies in mitochondrial DNA compromise the survival of yeast cells at critically high temperatures. Microbiol. Res. 2014; 169:185–195. 23890722 10.1016/j.micres.2013.06.011

[48] Lahtvee P.-J. , Kumar R., Hallström B.M., Nielsen J. Adaptation to different types of stress converge on mitochondrial metabolism. Mol. Biol. Cell. 2016; 27:2505–2514. 27307591 10.1091/mbc.E16-03-0187 PMC4966989

[49] Backes S. , Bykov Y.S., Flohr T., Räschle M., Zhou J., Lenhard S., Krämer L., Mühlhaus T., Bibi C., Jann C. et al. The chaperone-binding activity of the mitochondrial surface receptor Tom70 protects the cytosol against mitoprotein-induced stress. Cell Rep. 2021; 35:108936. 33826901 10.1016/j.celrep.2021.108936 PMC7615001

[50] Krämer L. , Dalheimer N., Räschle M., Storchová Z., Pielage J., Boos F., Herrmann J.M. MitoStores: chaperone-controlled protein granules store mitochondrial precursors in the cytosol. EMBO J. 2023; 42:e112309. 36704946 10.15252/embj.2022112309 PMC10068336

[51] Gardner R.G. , Swarbrick G.M., Bays N.W., Cronin S.R., Wilhovsky S., Seelig L., Kim C., Hampton R.Y Endoplasmic reticulum degradation requires lumen to cytosol signaling: transmembrane control of Hrd1p by Hrd3p. J. Cell Biol. 2000; 151:69–82. 11018054 10.1083/jcb.151.1.69 PMC2189800

[52] Cohen M. , Stutz F., Belgareh N., Haguenauer-Tsapis R., Dargemont C. Ubp3 requires a cofactor, Bre5, to specifically de-ubiquitinate the COPII protein, Sec23. Nat. Cell Biol. 2003; 5:661–667. 12778054 10.1038/ncb1003

[53] Müller M. , Kötter P., Behrendt C., Walter E., Scheckhuber C.Q., Entian K.-D., Reichert A.S. Synthetic quantitative array technology identifies the Ubp3-Bre5 deubiquitinase complex as a negative regulator of mitophagy. Cell Rep. 2015; 10:1215–1225. 25704822 10.1016/j.celrep.2015.01.044

[54] Penninckx M.J. An overview on glutathione in *Saccharomyces* versus non-conventional yeasts. FEMS Yeast Res. 2002; 2:295–305. 12702279 10.1016/S1567-1356(02)00081-8

[55] Lee J.-C. , Straffon M.J., Jang T.-Y., Higgins V.J., Grant C.M., Dawes I.W. The essential and ancillary role of glutathione in Saccharomyces cerevisiae analysed using a grande gsh1 disruptant strain. FEMS Yeast Res. 2001; 1:57–65. 12702463 10.1111/j.1567-1364.2001.tb00013.x

[56] Chen T.-H. , Wang H.-C., Chang C.-J., Lee S.-Y. Mitochondrial glutathione in cellular redox homeostasis and disease manifestation. Int. J. Mol. Sci. 2024; 25:1314. 38279310 10.3390/ijms25021314 PMC10816320

[57] Calahan D. , Dunham M., DeSevo C., Koshland D.E. Genetic analysis of desiccation tolerance in *Saccharomyces cerevisiae*. Genetics. 2011; 189:507–519. 21840858 10.1534/genetics.111.130369 PMC3189811

[58] Merico A. , Sulo P., Piškur J., Compagno C. Fermentative lifestyle in yeasts belonging to the *Saccharomyces* complex. FEBS J. 2007; 274:976–989. 17239085 10.1111/j.1742-4658.2007.05645.x

[59] Freel K.C. , Friedrich A., Schacherer J. Mitochondrial genome evolution in yeasts: an all-encompassing view. FEMS Yeast Res. 2015; 15:fov023. 25969454 10.1093/femsyr/fov023

[60] De Vries S. , Van Witzenburg R., Grivell L.A., Marres C.A. Primary structure and import pathway of the rotenone-insensitive NADH-ubiquinone oxidoreductase of mitochondria from *Saccharomyces cerevisiae*. Eur. J. Biochem. 1992; 203:587–592. 1735444 10.1111/j.1432-1033.1992.tb16587.x

[61] de Vries S. , Marres C.A. The mitochondrial respiratory chain of yeast. Structure and biosynthesis and the role in cellular metabolism. Biochim. Biophys. Acta. 1987; 895:205–239. 2849479 10.1016/s0304-4173(87)80003-4

[62] Marcet-Houben M. , Marceddu G., Gabaldón T. Phylogenomics of the oxidative phosphorylation in fungi reveals extensive gene duplication followed by functional divergence. BMC Evol. Biol. 2009; 9:295. 20025735 10.1186/1471-2148-9-295 PMC2803194

[63] Krause D.J The evolution of anaerobic growth in *Saccharomycotina* yeasts. Yeast. 2023; 40:395–400. 37526396 10.1002/yea.3890

[64] Dashko S. , Zhou N., Compagno C., Piškur J. Why, when, and how did yeast evolve alcoholic fermentation?. FEMS Yeast Res. 2014; 14:826–832. 24824836 10.1111/1567-1364.12161 PMC4262006

[65] Espinosa-Cantú A. , Ascencio D., Barona-Gómez F., DeLuna A. Gene duplication and the evolution of moonlighting proteins. Front. Genet. 2015; 6:227. 26217376 10.3389/fgene.2015.00227 PMC4493404

[66] Singh N. , Bhalla N. Moonlighting proteins. Annu. Rev. Genet. 2020; 54:265–285. 32870732 10.1146/annurev-genet-030620-102906

[67] Gupta M.N. , Uversky V.N. Moonlighting enzymes: when cellular context defines specificity. Cell. Mol. Life Sci. 2023; 80:130. 37093283 10.1007/s00018-023-04781-0 PMC11073002

[68] Veiga A. , Arrabaça J.D., Loureiro-Dias M.C. Cyanide-resistant respiration is frequent, but confined to yeasts incapable of aerobic fermentation. FEMS Microbiol. Lett. 2000; 190:93–97. 10981696 10.1111/j.1574-6968.2000.tb09268.x

[69] Anderson F.M. , Visser N.D., Amses K.R., Hodgins-Davis A., Weber A.M., Metzner K.M., McFadden M.J., Mills R.E., O’Meara M.J., James T.Y. et al. *Candida albicans* selection for human commensalism results in substantial within-host diversity without decreasing fitness for invasive disease. PLoS Biol. 2023; 21:e3001822. 37205709 10.1371/journal.pbio.3001822 PMC10234564

[70] Alves R. , Barata-Antunes C., Casal M., Brown A.J.P., Van Dijck P., Paiva S. Adapting to survive: how *Candida* overcomes host-imposed constraints during human colonization. PLoS Pathog. 2020; 16:e1008478. 32437438 10.1371/journal.ppat.1008478 PMC7241708

[71] Rodaki A. , Bohovych I.M., Enjalbert B., Young T., Odds F.C., Gow N.A.R., Brown A.J.P. Glucose promotes stress resistance in the fungal pathogen *Candida albicans*. Mol. Biol. Cell. 2009; 20:4845–4855. 19759180 10.1091/mbc.E09-01-0002 PMC2777113

[72] Hagman A. , Säll T., Compagno C., Piskur J. Yeast “make-accumulate-consume” life strategy evolved as a multi-step process that predates the whole genome duplication. PLoS One. 2013; 8:e68734. 23869229 10.1371/journal.pone.0068734 PMC3711898

[73] Gomes F. , Tahara E.B., Busso C., Kowaltowski A.J., Barros M.H. nde1 deletion improves mitochondrial DNA maintenance in *Saccharomyces cerevisiae* coenzyme Q mutants. Biochem. J. 2013; 449:595–603. 23116202 10.1042/BJ20121432

[74] Busso C. , Tahara E.B., Ogusucu R., Augusto O., Ferreira-Junior J.R., Tzagoloff A., Kowaltowski A.J., Barros M.H *Saccharomyces cerevisiae* coq10 null mutants are responsive to antimycin A. FEBS J. 2010; 277:4530–4538. 20875086 10.1111/j.1742-4658.2010.07862.x PMC3155804

[75] Bennett N.K. , Lee M., Orr A.L., Nakamura K. Systems-level analyses dissociate genetic regulators of reactive oxygen species and energy production. Proc. Natl. Acad. Sci. U.S.A. 2024; 121:e2307904121. 38207075 10.1073/pnas.2307904121 PMC10801874

[76] Jakubke C. , Roussou R., Maiser A., Schug C., Thoma F., Bunk D., Hörl D., Leonhardt H., Walter P., Klecker T. et al. Cristae-dependent quality control of the mitochondrial genome. Sci. Adv. 2021; 7:eabi8886. 34516914 10.1126/sciadv.abi8886 PMC8442932

[77] Kondadi A.K. , Anand R., Reichert A.S. Functional interplay between cristae biogenesis, mitochondrial dynamics and mitochondrial DNA integrity. Int. J. Mol. Sci. 2019; 20:4311. 31484398 10.3390/ijms20174311 PMC6747513

[78] Chapman J. , Ng Y.S., Nicholls T.J. The maintenance of mitochondrial DNA integrity and dynamics by mitochondrial membranes. Life. 2020; 10:164. 32858900 10.3390/life10090164 PMC7555930

[79] Needs H.I. , Protasoni M., Henley J.M., Prudent J., Collinson I., Pereira G.C. Interplay between mitochondrial protein import and respiratory complexes assembly in neuronal health and degeneration. Life. 2021; 11:432. 34064758 10.3390/life11050432 PMC8151517

[80] Kreimendahl S. , Rassow J. The mitochondrial outer membrane protein Tom70-mediator in protein traffic, membrane contact sites and innate immunity. Int. J. Mol. Sci. 2020; 21:7262. 33019591 10.3390/ijms21197262 PMC7583919

[81] Liu Q. , Chang C.E., Wooldredge A.C., Fong B., Kennedy B.K., Zhou C. Tom70-based transcriptional regulation of mitochondrial biogenesis and aging. eLife. 2022; 11:e75658. 35234609 10.7554/eLife.75658 PMC8926401

[82] Liu Q. , Fong B., Yoo S., Unruh J.R., Guo F., Yu Z., Chen J., Si K., Li R., Zhou C. Nascent mitochondrial proteins initiate the localized condensation of cytosolic protein aggregates on the mitochondrial surface. Proc. Natl Acad. Sci. U.S.A. 2023; 120:e2300475120. 37494397 10.1073/pnas.2300475120 PMC10401023

[83] Kemmeren P. , Sameith K., van de Pasch L.A.L., Benschop J.J., Lenstra T.L., Margaritis T., O’Duibhir E., Apweiler E., van Wageningen S., Ko C.W. et al. Large-scale genetic perturbations reveal regulatory networks and an abundance of gene-specific repressors. Cell. 2014; 157:740–752. 24766815 10.1016/j.cell.2014.02.054

[84] Spivakov M. Spurious transcription factor binding: non-functional or genetically redundant?. Bioessays. 2014; 36:798–806. 24888900 10.1002/bies.201400036 PMC4230394

[85] MacIsaac K.D. , Wang T., Gordon D.B., Gifford D.K., Stormo G.D., Fraenkel E. An improved map of conserved regulatory sites for *Saccharomyces cerevisiae*. BMC Bioinformatics. 2006; 7:113. 16522208 10.1186/1471-2105-7-113 PMC1435934

[86] Hu Z. , Killion P.J., Iyer V.R Genetic reconstruction of a functional transcriptional regulatory network. Nat. Genet. 2007; 39:683–687. 17417638 10.1038/ng2012

