# Supplementary material for: Protein moonlighting by a target gene dominates phenotypic divergence of the Sef1 transcriptional regulatory network in yeasts
Source: Nucleic Acids Res. 2024 Nov 20;52(22):13914–30. doi: 10.1093/nar/gkae1147 (PMC11662654; doi:10.1093/nar/gkae1147)
Supplement: gkae1147_Supplemental_Files [file gkae1147_supplemental_files.zip › Supplementary files_R2_20241009.pdf]

Protein moonlighting by a target gene dominates phenotypic divergence of the Sef1 transcriptional regulatory network in yeasts

Po-Chen Hsu[1]*, Tzu-Chiao Lu[2,3], Po-Hsiang Hung[4], and Jun-Yi Leu[1]*

1. Institute of Molecular Biology, Academia Sinica, Taipei, Taiwan, ROC

2. Huffington Center on Aging, Baylor College of Medicine, Houston, TX 77030, USA.

3. Department of Molecular and Human Genetics, Baylor College of Medicine, Houston, TX 77030, USA.

4. Department of Genetics, Stanford University Medical School, Stanford, California 94305, USA

* To whom correspondence should be addressed:

Po-Chen Hsu

Institute of Molecular Biology, Academia Sinica, 128 Sec.2, Academia Road, Nankang, Taipei 115, Taiwan

Email: godshi2006@gmail.com

Phone: 886-2-27899216

Jun-Yi Leu

Institute of Molecular Biology, Academia Sinica, 128 Sec.2, Academia Road, Nankang, Taipei 115, Taiwan

Email: jleu@imb.sinica.edu.tw

Phone: 886-2-26519574

Supplementary Figures

(A)

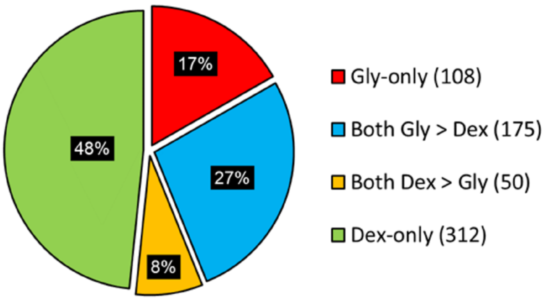

(B)

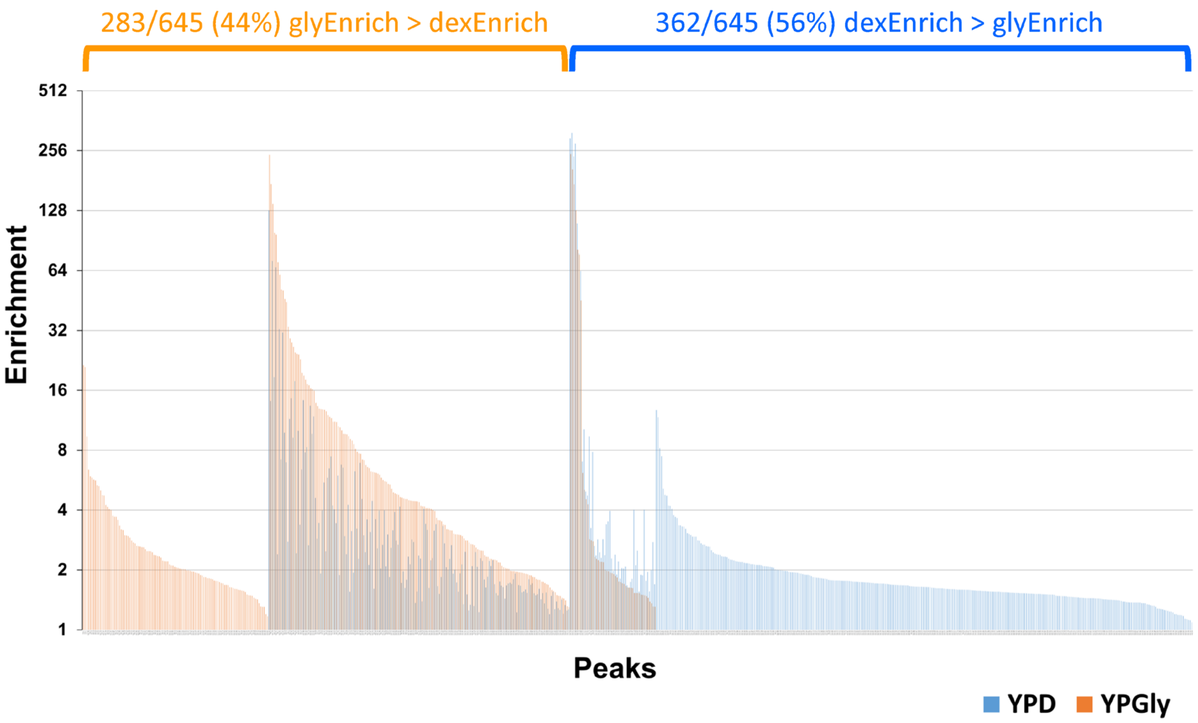

(C)

PeakN	TCA genes	dexEnrichAve	glyEnrichAve
Peak710	*ACO1*	239.23	173.39
Peak114	*ACO2*	1.68	0.00
Peak_641	*CIT1*	1.67	1.94
Peak_159	*CIT1*	2.25	0.00
Peak_676	*CIT2*	1.39	1.49
Peak_341	*CIT3*	3.10	6.27
Peak_13	*FUM1*	6.95	44.42
Peak_308	*IDH1*	276.90	128.46
Peak_73	*IDH1*	9.36	2.86
Peak_758	*IDH2*	294.31	245.43
Peak_66	*IDP2*	3.98	12.84
Peak_22	*MDH1*	1.55	9.11
Peak_10	*MDH2*	2.66	16.98
Peak_423	*SDH1b*	1.98	0.00

(D)

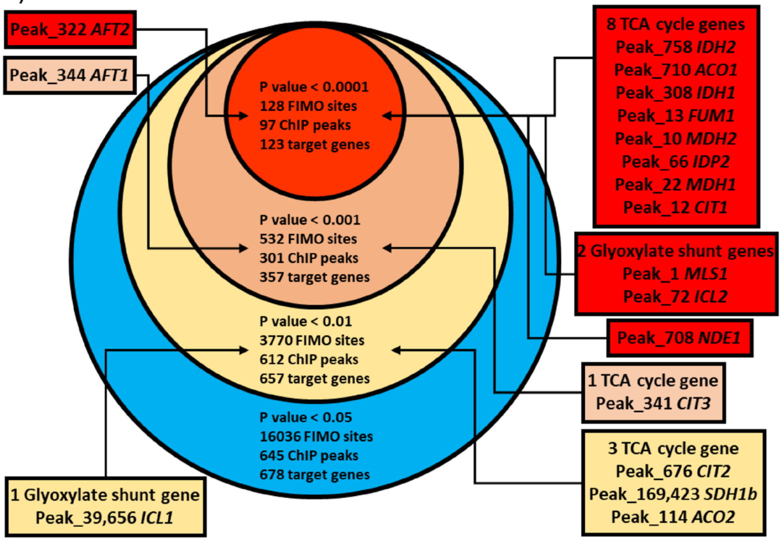

Figure S1. ChIP-seq analyses for ScSef1 under both YPD and YPGly conditions.

(A) The number and frequency of each group of Sef1-bound peaks called from ChIP-seq. Each group is classified according to the enrichment values (glyEnrich and dexEnrich). The peaks from each set were identified from convergent peaks among three biological repeats. A total of 645 (108 + 175 + 50 +312) peaks were identified.

(B) Peak ranking according to ChIP-seq enrichment values. All 645 peaks (Peak_N, x-axis) are ranked based on enrichment values (y-axis) in descending order. For each peak, two enrichment values have been paired and are depicted (dexEnrich：YPD (in blue); glyEnrich：YPGly (in orange)). If a peak was not called in all biological replicates or was absent from one of the biological replicates in either of two conditions, the enrichment value of the peak under that condition was assigned as "zero". Furthermore, peak sets have been sorted according to two criteria：glyEnrich > dexEnrich (283 peaks) and dexEnrich > glyEnrich (362 peaks). From left to right along the x-axis, the peaks are listed in the following order: glyEnrich-only, glyEnrich > dexEnrich, dexEnrich > glyEnrich, and dexEnrich-only.

(C) The ScSef1-targeted TCA cycle genes according to ChIP-seq. The ChIP enrichment values (from wild-type Sef1 ChIP-seq data, Table S1) of peaks that target each gene under the YPD and YPGly conditions (dexEnrich and glyEnrich, respectively) are included in the table. The highly enriched target genes (including *ACO1*, *IDH1*, and *IDH2*) are highlighted in red. If a peak was not called in all three biological replicates or was absent from one of the biological replicates in either of two conditions, the enrichment value of the peak under that condition was assigned as "zero".

(D) Motif re-scanning of ScSef1 target genes by FIMO. Scanning of 645 ChIP peaks for occurrences of the ScSef1 binding motif. Scanning results (numbers of peaks and motif sites) sorted according to different p-value cut-offs (<0.05 to <0.0001) are shown in the stacked Venn diagram. The distributions of 12 peaks targeting TCA cycle genes, 4 peaks targeting glyoxylate shunt genes, 1 peak for *NDE1*, and 2 peaks for the paralogous iron-responsive

regulators *AFT2* and *AFT1*, are indicated on the diagram.

(A)

	TOP50 peaks<br>High affinity (Enrich ≥ 10)	51~645 peaks<br>Low affinity (Enrich < 10)	*Row total*
TCA	7	5	*12*
Non-TCA	43	590	*633*
Column total	*50*	*595*	*645 (Grand total)*

χ^2 (1, N = 645) = 43.7473, $p < 0.00001$

(B)

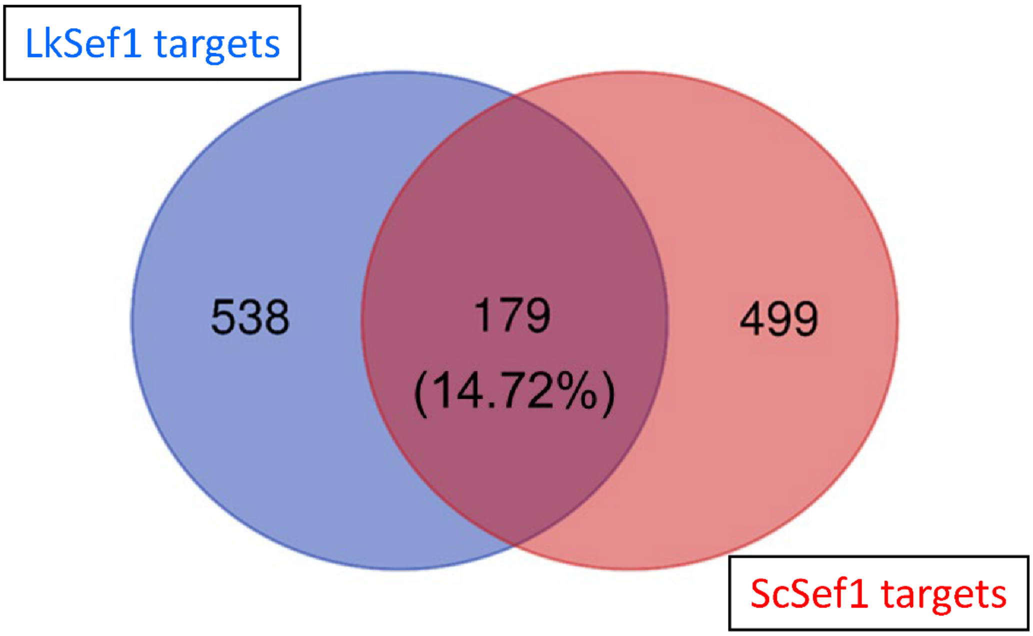

(C)

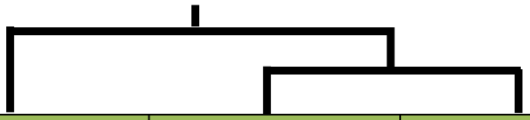

**TCA cycle genes**	***C. albicans***	***L. kluyveri***	***S. cerevisiae***
Sef1-binding on the promoter	7/21 = 33.3%	18/21 = 85.7%	12/28 = 42.9%
Sef1-dependent expression	0	6/21 = 28.6%	1/28 = 3.6%
Status of Sef1-binding mediated regulation	Non-functional	Moderate regulation	Weak regulation

Figure S2. Sef1-mediated vestigial regulation of TCA cycle regulons in *S. cerevisiae*.

(A) A contingency table displaying frequencies of ScSef1 ChIP targets classified into TCA

cycle/non-TCA cycle genes and high-/low-affinity targets was used for the Chi-square test of independence. The high-affinity targets are defined as Sef1 targets with ChIP enrichment fold-changes (Enrich) ≥10 under the YPGly condition (i.e., TOP50 peaks), whereas the low-affinity targets are those with fold-changes <10 (i.e., the remaining 595 peaks). The online calculator (https://www.socscistatistics.com/tests/chisquare2/default2.aspx) for the Chi-square test was used. There is a significant association ($p < 0.00001$) between Sef1 binding affinity and TCA cycle genes, i.e., the TCA cycle is enriched in high-affinity Sef1 targets.

(B) Overlap in ChIP target genes of *L. kluyveri* Sef1 (blue) and *S. cerevisiae* Sef1 (red). There are 14.72% conserved targets among Sef1 targets of both species. Only conserved genes in both species were considered. Venn diagram areas are not proportional.

(C) Different levels of vestigial regulation mediated by Sef1 on TCA cycle genes in *C. albicans*, *L. kluyveri*, and *S. cerevisiae*. “Vestigial regulation” results from non-functional Sef1 binding (see Fig 2D). “Sef1-binding on the promoter” was determined by ChIP-seq. “Sef1-dependent expression” was determined by WT versus *sef1Δ* gene expression analyses. Relative to CaSef1, LkSef1 and ScSef1 moderately and weakly regulate TCA cycle genes, respectively.

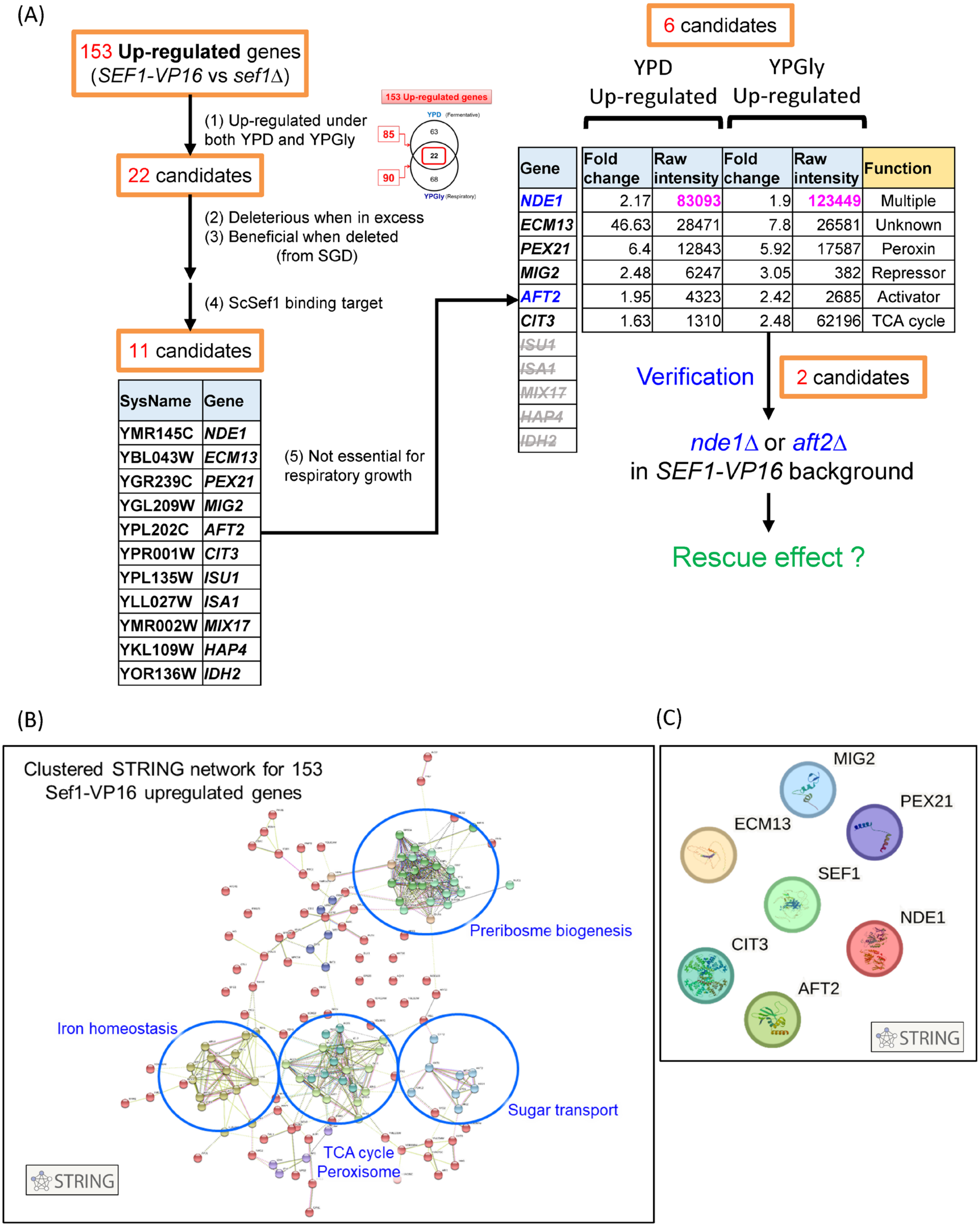
(A)
153 Up-regulated genes
(SEF1-VP16 vs sef1Δ)
(1) Up-regulated under both YPD and YPGly
153 Up-regulated genes
YPD (Fermentative)
85
63
22
68
90
YPGly (Respiratory)
22 candidates
(2) Deleterious when in excess
(3) Beneficial when deleted
(from SGD)
(4) ScSef1 binding target
11 candidates
SysName Gene
YMR145C NDE1
YBL043W ECM13
YGR239C PEX21
YGL209W MIG2
YPL202C AFT2
YPR001W CIT3
YPL135W ISU1
YLL027W ISA1
YMR002W MIX17
YKL109W HAP4
YOR136W IDH2
(5) Not essential for respiratory growth
6 candidates
YPD Up-regulated
YPGly Up-regulated
Gene | Fold change | Raw intensity | Fold change | Raw intensity | Function
NDE1 | 2.17 | 83093 | 1.9 | 123449 | Multiple
ECM13 | 46.63 | 28471 | 7.8 | 26581 | Unknown
PEX21 | 6.4 | 12843 | 5.92 | 17587 | Peroxin
MIG2 | 2.48 | 6247 | 3.05 | 382 | Repressor
AFT2 | 1.95 | 4323 | 2.42 | 2685 | Activator
CIT3 | 1.63 | 1310 | 2.48 | 62196 | TCA cycle
ISU1
ISA1
MIX17
HAP4
IDH2
Verification
2 candidates
nde1Δ or aft2Δ
in SEF1-VP16 background
Rescue effect ?
(B)
Clustered STRING network for 153 Sef1-VP16 upregulated genes
Preribosme biogenesis
Iron homeostasis
Sugar transport
TCA cycle
Peroxisome
STRING
(C)
MIG2
PEX21
ECM13
SEF1
NDE1
CIT3
AFT2
STRING

Figure S3. *NDE1* and *AFT2* are the causal gene candidates responsible for Sef1-mediated phenotypic outcomes in *S. cerevisiae*.

(A) Workflow of direct causal gene candidate selection. The candidates were selected from 153 up-regulated genes in response to Sef1 hyperactivity in comparison to *sef1*Δ. After filtering according to the five criteria shown on the left panel to generate the final list of six candidates (right panel), the most abundant *NDE1* in the *SEF1-VP16* strain was first chosen for verification. *AFT2* was also selected due to its function as a transcription activator (also see Fig S4A). These two candidates were then deleted from the *SEF1-VP16* strain to assess the individual or combined rescue effects. *ECM13* and *PEX21* were not chosen due to unclear and redundant functions in the presence of the ohnolog *PEX18*, respectively. *MIG2* was not selected because of its negative role in gene expression, and *CIT3* was also excluded owing to its low abundance under the YPD condition.

(B) Clustered STRING interaction network of 153 Sef1-VP16-up-regulated genes. The full STRING network was built using the online STRING database (https://string-db.org/). The edges represent "evidence", including from databases, experiments, co-expression, and text mining. K-means clustering was applied to group the network into 10 clusters. Clusters containing genes enriched in specific GO terms are highlighted. Functional enrichments in the network were performed using the default "STRING Analysis" function.

(C) STRING network of *SEF1* with the six causal gene candidates, revealing no high-confidence interaction (edge) among them.

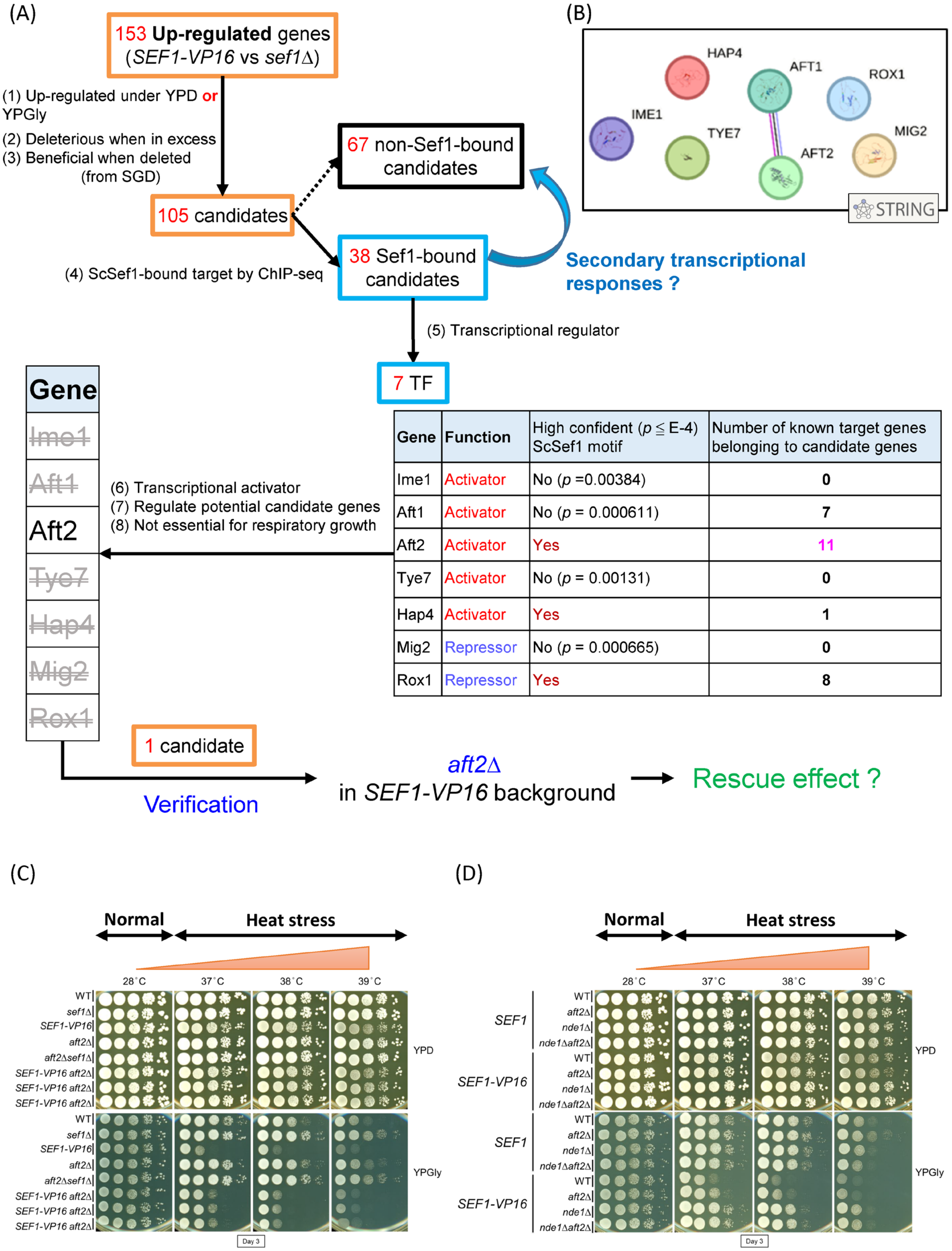

Gene	Function	High confident ($p \leq$ E-4) ScSef1 motif	Number of known target genes belonging to candidate genes
Ime1	Activator	No (p =0.00384)	0
Aft1	Activator	No (p = 0.000611)	7
Aft2	Activator	Yes	11
Tye7	Activator	No (p = 0.00131)	0
Hap4	Activator	Yes	1
Mig2	Repressor	No (p = 0.000665)	0
Rox1	Repressor	Yes	8

Figure S4. *AFT2* is the potential primary causal gene inducing the secondary responses in gene expression.

(A) Workflow of indirect causal gene candidate selection. The candidates were selected from 153 up-regulated genes in response to Sef1 hyperactivity relative to *sef1*Δ. After filtering according to the eight criteria shown in the flowchart, only *AFT2* was chosen for verification. *AFT2* is the only transcriptional activator that co-regulates multiple Sef1-induced genes with fitness effects and it is not required for respiratory growth (i.e., it is feasible to delete *AFT2* to assess its rescue effect under heat-stressed respiratory conditions) (also see Fig S3A).

(B) STRING network of seven TFs with the potential to be causal genes, revealing no high-confidence interaction (edge) among them, except for the paralogous iron-responsive activators Aft1 and Aft2, which exert partially redundant functions.

(C) Growth of the *SEF1-VP16 aft2*Δ mutants in response to YPD, YPGly, and heat stress in *S. cerevisiae*. All plates were incubated for 3 days. Three independent *SEF1-VP16 aft2*Δ clones were tested. Deletion of *AFT2* can marginally rescue the growth defects generated by hyperactive Sef1.

(D) Growth of the *SEF1-VP16 nde1*Δ*aft2*Δ mutants in response to YPD, YPGly, and heat stress in *S. cerevisiae*. All plates were incubated for 3 days. There is no clear additive rescue effect when both *AFT2* and *NDE1* are deleted.

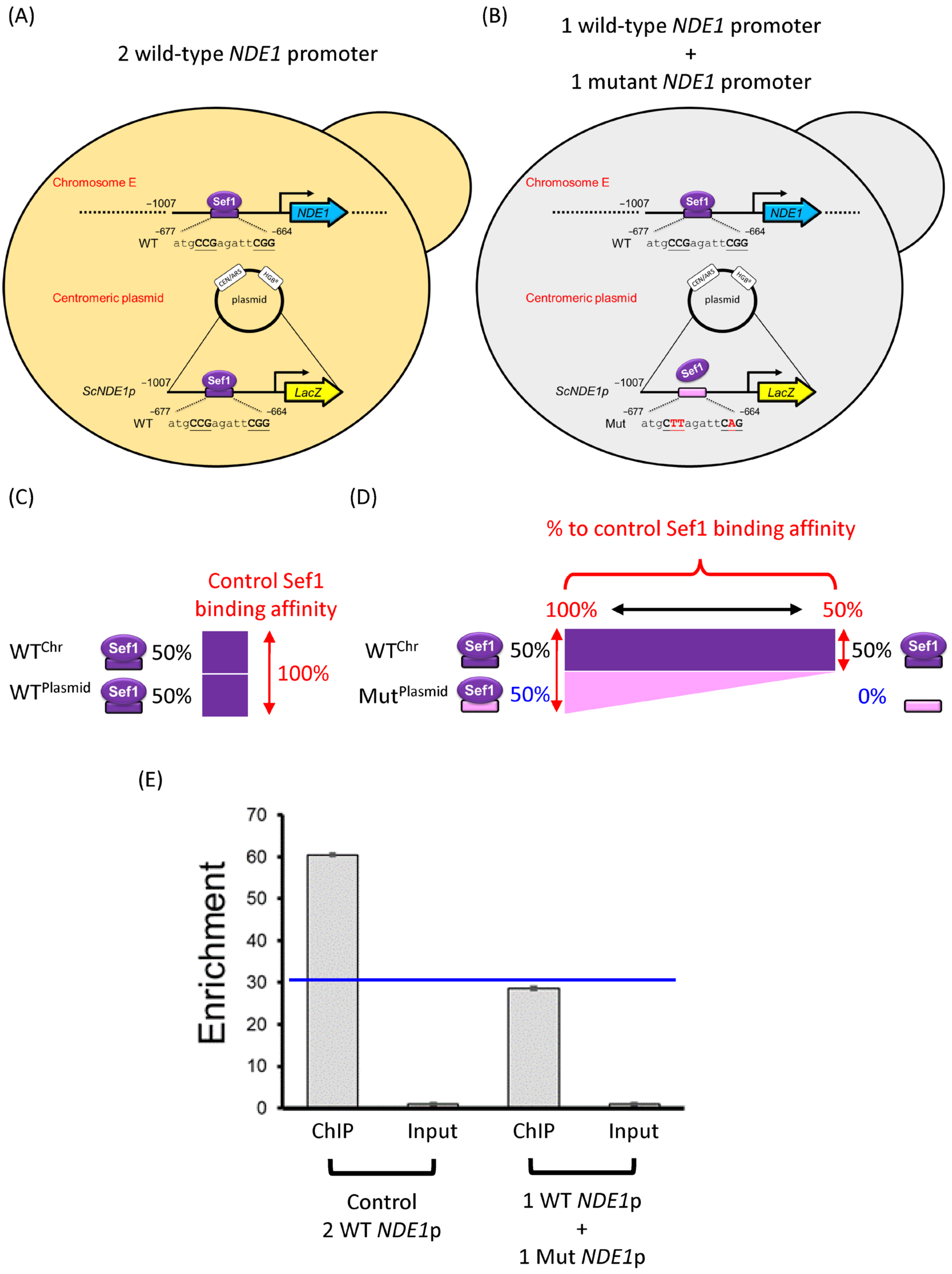
(A)
2 wild-type NDE1 promoter
Chromosome E
−1007
Sef1
NDE1
−677
−664
WT atgCCGagattCGG
Centromeric plasmid
CEN/ARS
plasmid
ScNDE1p
−1007
Sef1
LacZ
−677
−664
WT atgCCGagattCGG
(B)
1 wild-type NDE1 promoter
+
1 mutant NDE1 promoter
Chromosome E
−1007
Sef1
NDE1
−677
−664
WT atgCCGagattCGG
Centromeric plasmid
CEN/ARS
plasmid
Sef1
ScNDE1p
−1007
LacZ
−677
−664
Mut atgCTTagattCAG
(C)
Control Sef1 binding affinity
WTChr
Sef1
50%
WTPlasmid
Sef1
50%
100%
(D)
% to control Sef1 binding affinity
100%
50%
WTChr
Sef1
50%
MutPlasmid
Sef1
50%
50%
Sef1
0%
(E)
Enrichment
70
60
50
40
30
20
10
0
ChIP
Input
ChIP
Input
Control
2 WT NDE1p
1 WT NDE1p
+
1 Mut NDE1p

Figure S5. The intact CCGnnnnnCGGnnn consensus motif is required for ScSef1 binding.

(A) The control ScSef1-TAP ChIP strain carrying one wild-type copy of *NDE1* promoter on the chromosome and the other wild-type copy of *NDE1* promoter on the centromeric plasmid.

(B) The mutant ScSef1-TAP ChIP strain carrying one wild-type copy of *NDE1* promoter on the chromosome and one mutant copy of *NDE1* promoter with the mutated Sef1-binding motif on the centromeric plasmid.

(C) The maximal Sef1 binding affinity to the *NDE1* promoter in the control ScSef1-TAP ChIP strain is the sum of its binding affinity to the two wild-type promoter copies.

(D) The Sef1 binding affinity to the *NDE1* promoter in the mutant ScSef1-TAP ChIP strain relative to the control strain is determined by its binding affinity to the mutant promoter. The detected relative binding affinity (% to the control) may vary between 100% (if Sef1 shows the same binding affinity to both wild-type and mutant motifs) and ≤50% (if Sef1 cannot bind to the mutant motif).

(E) The sum of ScSef1 ChIP-binding affinity to two *NDE1* promoters under the YPGly condition. The mutated motif abolished (i.e., caused ≤50% total binding relative to the control sample) the binding of Sef1. Both control (A) and mutant (B) strains were assayed. The qPCR primers recognizing DNA sequences flanking the Sef1 motif were used to detect Sef1 binding. The qPCR primers recognizing the telomere end at the right arm of chromosome 6 were used as the unbound background for normalization (ΔCt). The fold enrichment of ChIP relative to each input was calculated using the $2^{-\Delta\Delta Ct}$ method. The blue line indicates the 50% fold enrichment value of the control (2 WT *NDE1*p). WT: the wild-type *NDE1*p promoter; Mut: the mutant *NDE1* promoter with the mutated Sef1-binding motif.

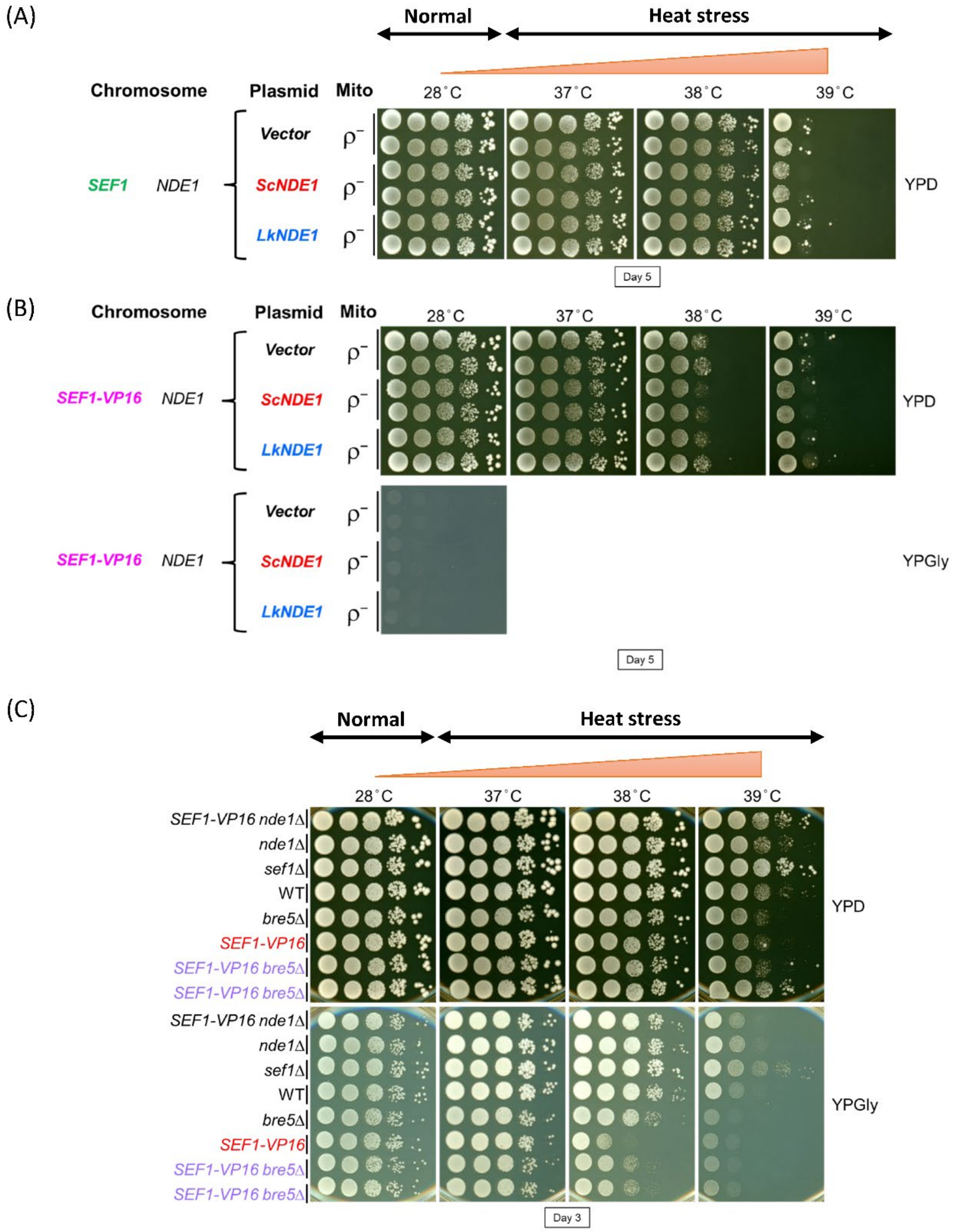

Figure S6. Nde1-induced cytotoxicity in response to mitochondrial defects is not the major mechanism responsible for the Sef1-regulated fitness effects.

(A) Growth of the ectopic *NDE1* strains in response to YPD and heat stress in *SEF1* petite cells.

(B) Growth of the ectopic *NDE1* strains in response to YPD, YPGly, and heat stress in *SEF1-VP16* petite cells.

For (A) and (B), the ectopic *NDE1* was expressed under the control of its native promoter on a centromeric plasmid selected by HGB in *S. cerevisiae* petite cells. Relative to vector-only controls, additional expression of *ScNDE1* only very slightly reduced cell growth, whereas *LkNDE1* did not affect the growth of the petite cells. The petite (ρ^-) phenotype was confirmed by growth on YPGly plates. Two independent clones each were tested.

(C) Growth of the *SEF1-VP16 bre5*Δ mutants in response to YPD, YPGly, and heat stress in *S. cerevisiae*. All plates were incubated for 3 days. Two independent *SEF1-VP16 bre5*Δ clones were tested. Abolishing the Bre5 function increases cellular mitophagy, thereby potentially eliminating bad mitochondria more efficiently. Deletion of *BRE5* partially rescued the defective growth caused by Sef1 hyperactivity, despite this rescue effect seemingly being temperature-specific.

(A)

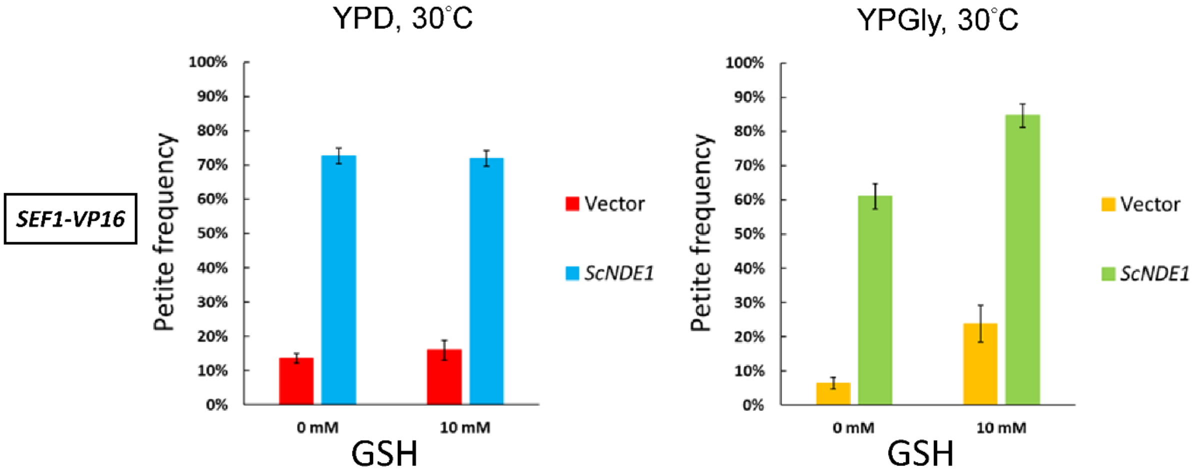

(B)

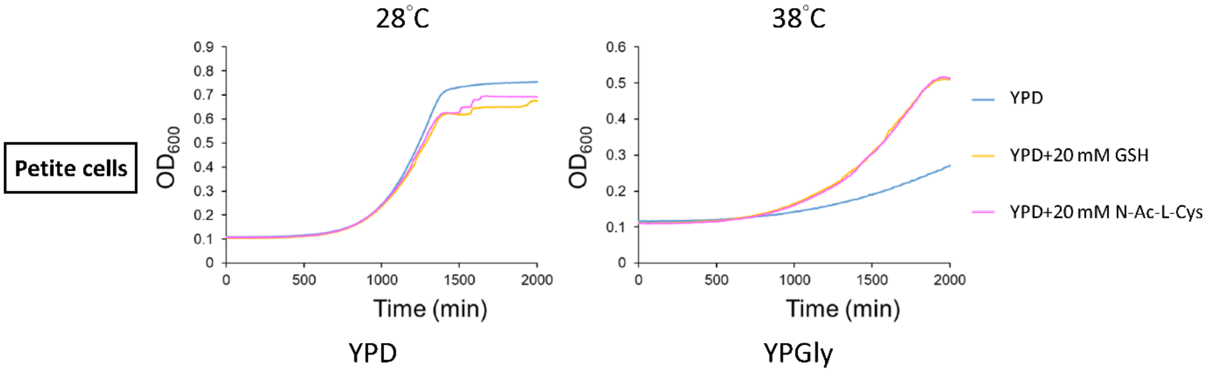

Figure S7. Glutathione supplementation does not inhibit Nde1-induced petite formation but directly promotes growth of petite cells under heat stress.

(A) The effects of glutathione (GSH) on petite formation. Petite formation was induced by Sef1 hyperactivity and ectopic *ScNDE1* on a centromeric plasmid selected by HGB. Petite cells were counted after 20 hours of growth at 30°C in YPD or YPGly, with or without 10 mM GSH. The petite frequency is displayed as mean ± SD from four to five technical repeats. GSH supplementation did not reduce petite frequency.

(B) Growth of petite cells in response to GSH and its precursor N-Acetyl-L-cysteine (N-Ac-L-Cys) supplementation in YPD. GSH or N-Ac-L-Cys replenishment markedly rescued the growth defects of petite cells under heat stress (38°C). The petite strains were

derived from wild-type strains with ectopic *ScNDE1* on a centromeric plasmid selected by HGB.

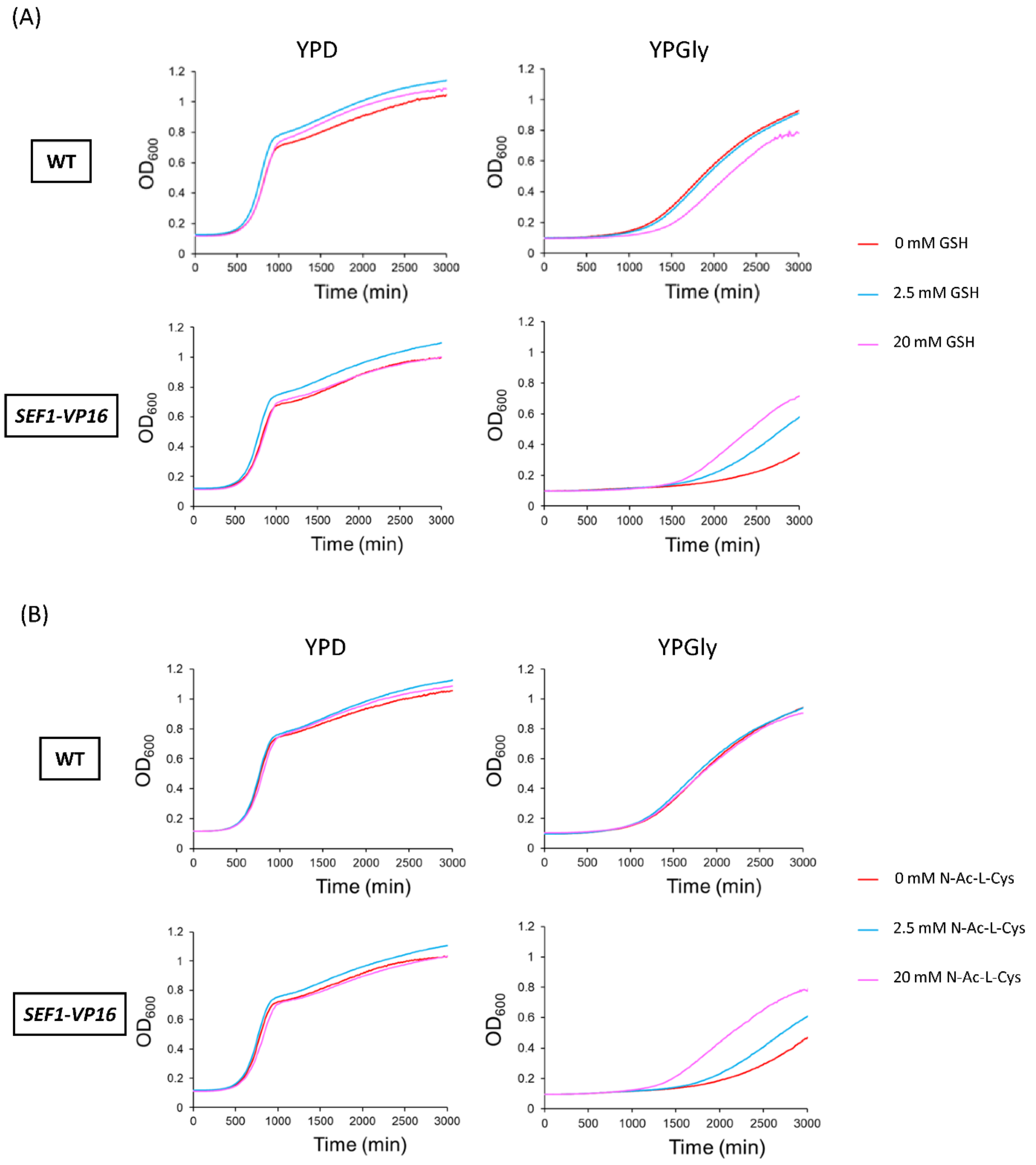

Figure S8. Growth of petite cells in the population determines ScSef1-mediated fitness.

(A) Growth of the wild-type and *SEF1-VP16* strains in response to GSH under heat stress (38°C).

(B) Growth of the wild-type and *SEF1-VP16* strains in response to N-Ac-L-Cys under heat stress (38°C).

For (A) and (B), GSH and N-Ac-L-Cys supplementation alleviated the growth defects of the *SEF1-VP16* strain in a concentration-dependent manner in YPGly but not in YPD and did not improve growth of the wild type.

(A)

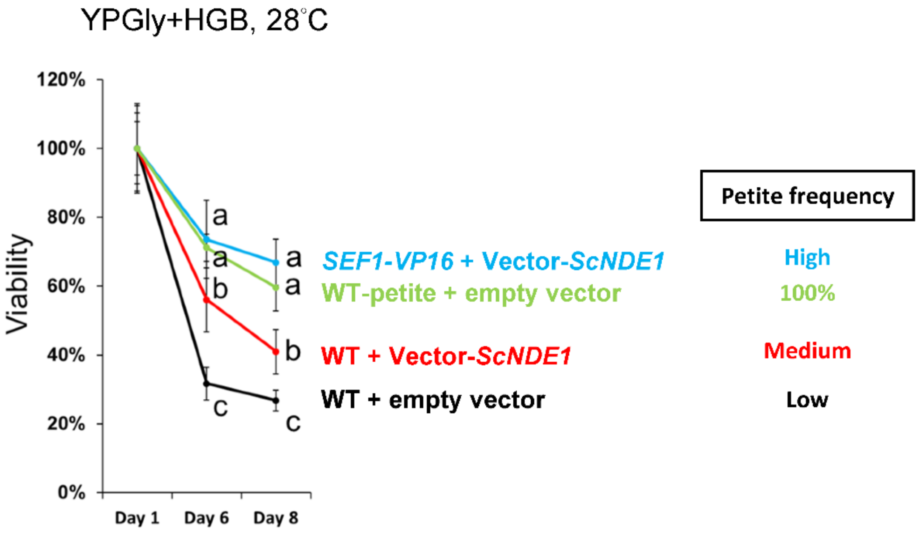

(B)

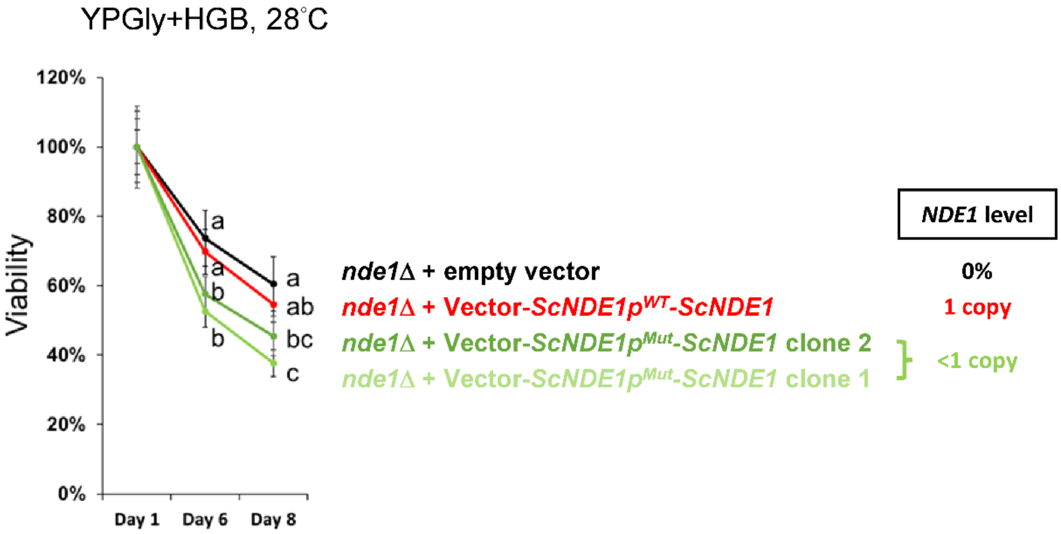

Figure S9. High levels of *NDE1* expression can provide condition-specific benefits.

(A) Viability of *S. cerevisiae* strains expressing different levels of plasmid-borne *NDE1* in steady respiratory culture. This condition favors the survival of non-dividing petite cells. The wild-type petite strain was used as a control culture of non-dividing cells. Compared to the non-dividing culture, survival rates increase with increased levels of *NDE1* expression.

(B) Viability of *S. cerevisiae* strains with or without Sef1-promoted *NDE1* expression from plasmids in steady respiratory culture. Compared to *nde1*Δ, wild-type *NDE1* levels reduced cell viability. However, insufficient *NDE1* expression caused by mutation of the Sef1 binding

motif (see Fig 3D) in the *NDE1* promoter reduced cell viability even more.

For (A) and (B), the cells were inoculated in YPGly with 300 μg/ml HGB and incubated at 28°C for 8 days. The viabilities at each indicated time point were measured by counting CFUs and are displayed as mean ± SD from six technical repeats. One-way ANOVA followed by Tukey's multiple comparisons post hoc test was performed on data from Day 6 and Day 8, with significant differences indicated by letters.

(A)

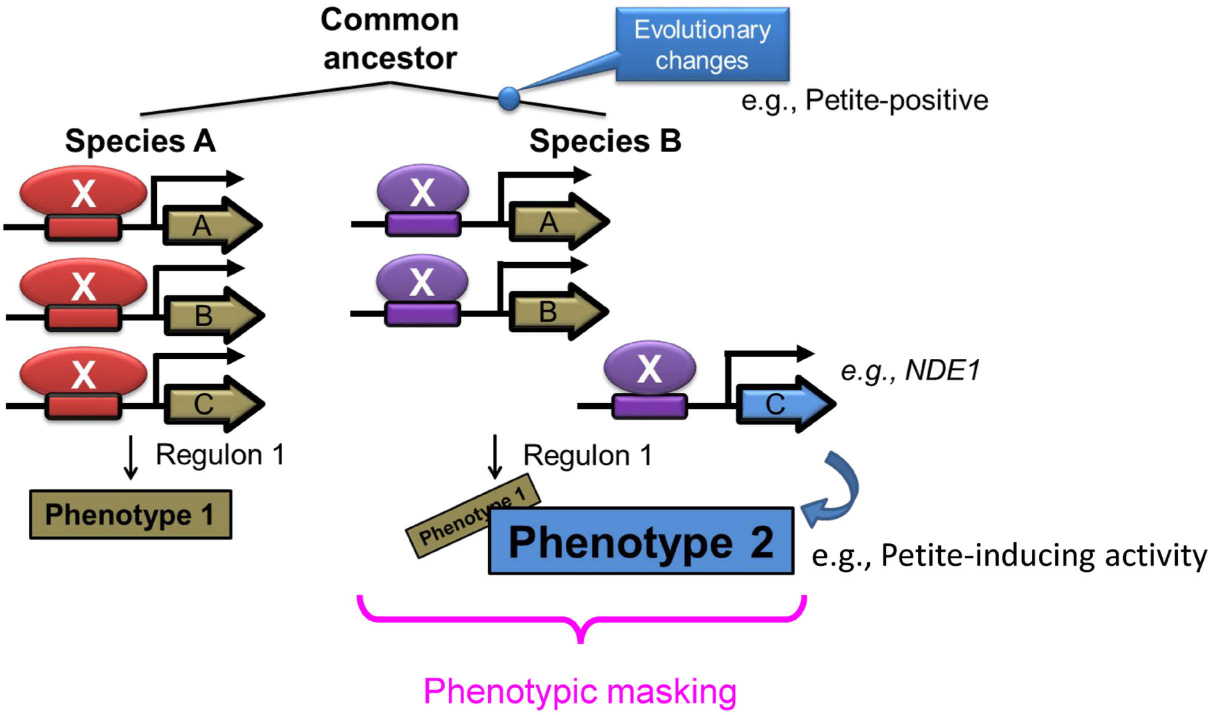

Common ancestor
Evolutionary changes
e.g., Petite-positive
Species A
Species B
X
A
B
C
e.g., NDE1
Regulon 1
Phenotype 1
Phenotype 2
e.g., Petite-inducing activity
Phenotypic masking

(B)

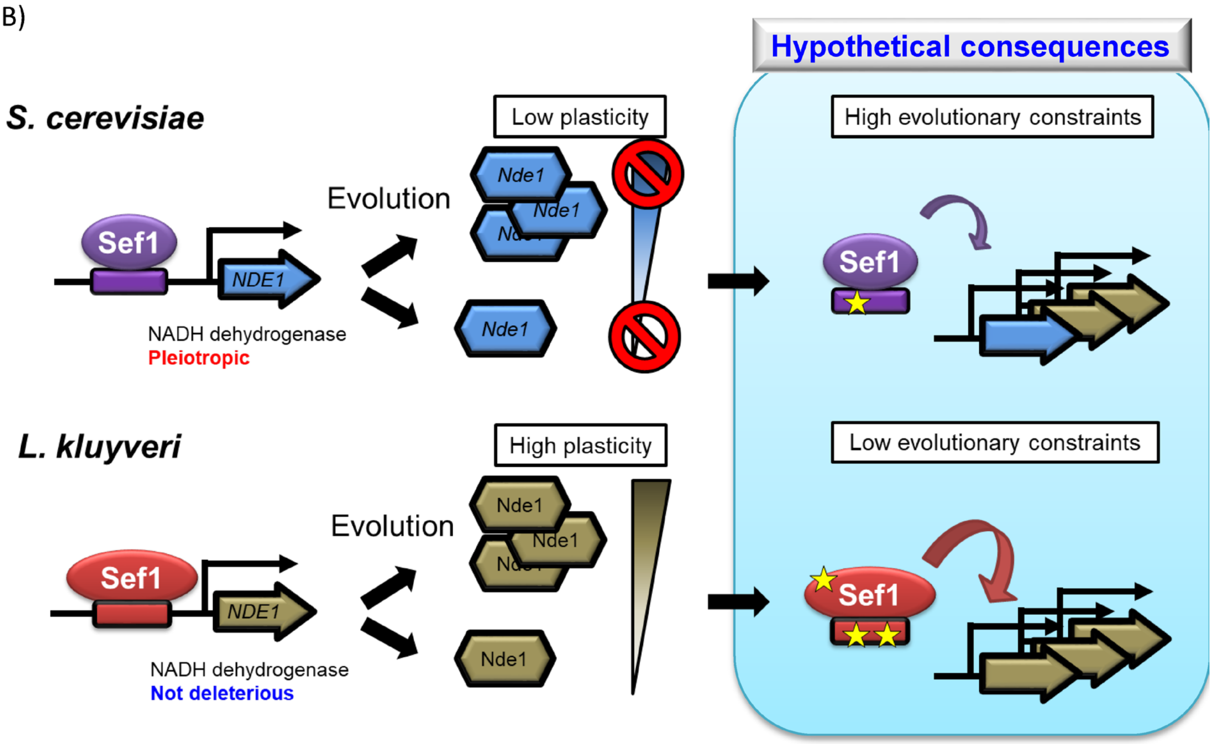

Hypothetical consequences
S. cerevisiae
Low plasticity
High evolutionary constraints
Evolution
Sef1
NDE1
Nde1
NADH dehydrogenase
Pleiotropic
L. kluyveri
High plasticity
Low evolutionary constraints
NADH dehydrogenase
Not deleterious

Figure S10. Phenotypic masking by Nde1 diverges the phenotypic outcome of the ScSef1 regulatory network in the TCA cycle-regulating branch.

(A) Phenotypic masking by the functional change of a strong-effect target gene (e.g., *ScNDE1*) leads to a pseudo-functional switch of the transcriptional regulator (e.g., ScSef1) without a comprehensive alteration of the major topology of the regulatory network (e.g., conserved Sef1-targeting to TCA cycle genes and *NDE*1 in both *L. kluyveri* and *S. cerevisiae*).

(B) Hypothetical consequences of the evolution of orthologous Sef1 regulatory networks. ScSef1 and ScNde1 exemplify a pleiotropic target gene encoding a moonlighting protein with both beneficial and deleterious functions that may constrain the future evolution of its transcriptional regulator due to the requirement to maintain flexible but steady target gene expression within a specific range. In contrast, evolution of LkSef1 may not suffer this evolutionary constraint due to its higher tolerance to the plastic expression of *LkNDE1*.

Supplementary Tables

Table S1. Complete ChIP-seq results of *S. cerevisiae* Sef1.

Table S2. Differentially expressed gene profiles in the early log-phase *sef1*Δ mutant compared to the wild type in YPD.

Table S3. Differentially expressed gene profiles in the early log-phase *sef1*Δ mutant compared to the wild type in YPGly.

Table S4. Differentially expressed gene profiles in the early log-phase hyperactive *SEF1-VP16* mutant compared to the wild type in YPD.

Table S5. Differentially expressed gene profiles in the early log-phase hyperactive *SEF1-VP16* mutant compared to the wild type in YPGly.

Table S6. Differentially expressed gene profiles in the early log-phase hyperactive *SEF1-VP16* mutant compared to the *sef1*Δ mutant in YPD.

Table S7. Differentially expressed gene profiles in the early log-phase hyperactive *SEF1-VP16* mutant compared to the *sef1*Δ mutant in YPGly.

Table S8. Genome resources.

Table S9. Lists of strains, plasmids, primers, media, and chemicals.
